# A dynamic directed transfer function for brain functional network-based feature extraction

**DOI:** 10.1186/s40708-022-00154-8

**Published:** 2022-03-18

**Authors:** Mingai Li, Na Zhang

**Affiliations:** 1 Faculty of Information Technology, Beijing University of Technology, Beijing, 100124 China; 2 Beijing Key Laboratory of Computational Intelligence and Intelligent System, Beijing, 100124 China; 3 Engineering Research Center of Digital Community, Ministry of Education, Beijing, 100124 China

**Keywords:** Directed transfer function, Feature extraction, Motor imagery electroencephalogram, Brain functional network, Graph theory

## Abstract

Directed transfer function (DTF) is good at characterizing the pairwise interactions from whole brain network and has been applied in discrimination of motor imagery (MI) tasks. Considering the fact that MI electroencephalogram signals are more non-stationary in frequency domain than in time domain, and the activated intensities of *α* band (8–13 Hz) and *β* band [13–30 Hz, with $\beta_{1}$(13–21 Hz) and $\beta_{2}$(21–30 Hz) included] have considerable differences for different subjects, a dynamic DTF (DDTF) with variable model order and frequency band is proposed to construct the brain functional networks (BFNs), whose information flows and outflows are further calculated as network features and evaluated by support vector machine. Extensive experiments are conducted based on a public BCI competition dataset and a real-world dataset, the highest recognition rate achieve 100% and 86%, respectively. The experimental results suggest that DDTF can reflect the dynamic evolution of BFN, the best subject-based DDTF appears in one of four frequency sub-bands (*α*, *β*, $\beta_{1} ,$ ${ }\beta_{2}$) for discrimination of MI tasks and is much more related to the current and previous states. Besides, DDTF is superior compared to granger causality-based and traditional feature extraction methods, the *t*-test and Kappa values show its statistical significance and high consistency as well.

## Introduction

Brain–computer interface (BCI) is a technology that allows people with healthy bodies or motor impairments to communicate with external environment through their brains’ activity without the assistance of peripheral nerves and muscles [1, 2]. Motor imagery (MI) refers to a mental process by which an individual rehearses or simulates a given action, for example, imaging the left or right hand movement without actually executing it. Motor imagery electroencephalography (MI-EEG) is commonly used in BCI owing to its advantages of high temporal resolution, high portability and few risks. Complex motor imagery can activate scattered areas of cortex, mainly including the somatosensory and somatomotor areas, and the brain activations vary with different MI tasks. MI-EEG is a multi-channel non-stationary and time–frequency signal with spatial distribution characteristic, and it shows obvious individual differences. The MI responsive frequency bands are not consistent for inter-/intra-subjects, this reflects the subject-based characteristics of MI-EEG. The MI-based BCI interprets mental activities by identifying EEG signals of different MI tasks, and realizes the control and exchange of information between the brain and the outside world. The key to improve recognition accuracy is how to effectively use these characteristics of MI-EEG for feature extraction [3].

In the past years, a variety of feature extraction methods have been used in classifying mental tasks. Common spatial pattern (CSP) is one of the most popular and efficient algorithms which is particularly renowned for the high classification rates, notably in BCI competitions [4–6]. However, the classical CSP also has its limitations which are sensitive to noise, overfitting and individual variability. To tackle these issues, a series of CSP-based methods have been proposed, including ‘analytic signal’-based CSP (ACSP) [6], correlation-based channel selection common spatial pattern (CCS-RCSP) [7], common complex-spatio spectral pattern (CCSSP) [8] and complex common spatial patterns (CCSPs) [9]. The variants of CSP methods make full of the multi-channel and spatial distribution characteristics of MI-EEG for feature extraction and achieve preferable classification accuracies. Regretfully, the information transfer and flow between channels have not received much attention. In fact, EEG researchers have tended to reveal that during performing even simple motor or cognitive tasks, many different functional areas widely scattered over the brain are mutually interconnected and exchange their information with one another, thus making it hard to isolate one or two regions where the activity takes place [10]. Physiologically speaking, when humans are involved in a motor task, our brains function as a complex network with the interactions of specialized, spatially distributed but functionally linked brain regions [11–15], contributing to the related brain functions. Therefore, it is an important strategy for ameliorating MI-EEG feature extraction to discover and use information flow between multi-channel EEG signals.

Recent years have witnessed that many researchers topologically characterize brain functional network by employing a mathematical framework called graph theory [16–18]. In graph theory, a network is defined as a graph formed by a set of nodes interconnected by edges [19]. Nodes in large-scale brain functional networks usually represent regions of interest (ROIs) or EEG electrodes, while links represent functional connectivity [17, 18, 20–22]. Generally, we can construct directed or undirected brain functional networks depending on adopting symmetric or asymmetric metrics of coupling between two signals. Anyway, in order to obtain abundant and exact properties, directed brain functional network is employed in this study. Granger causality (GC), as one of the commonly used techniques to construct a directed brain functional network, is aimed to illuminate casual temporal relations and directional nature of information flow for a pair of EEG channels. The basic notion of GC was originally conceived by Wiener [23], later adopted and formalized by Granger in the form of linear regression model. Granger’s causality concept has attracted a lot of people’s attention and has been extensively applied in the field of economics and neuroscience [24]. Since spectral properties are significant in biomedical signal analysis, extension of the concept to the frequency domain representation of time series was formulated by Geweke [25]. Although the above methods had lots of applications in many areas, it should be noted that they estimated directional information flow through bivariate approaches without using the whole covariance structure for a multivariate system. Unfortunately, it was reported that bivariate measures in certain cases could potentially give spurious connections and misleading results especially when some signals are fed from common channel sources, as is very likely in neurobiological systems [25].

To meet the demands, a full multivariate estimator, i.e., directed transfer function (DTF), was proposed to overcome the limitations of bivariate autoregressive methods and characterize directional connectivity as well as spectral properties of the interactions between any given pair of brain signals, and only one multivariate autoregressive (MVAR) model was required to be estimated simultaneously from all the EEG time recordings [26–28]. That is, all signals are regarded as members of one system and their mutual influences (not limited to pairwise connections) are considered. Subsequently, many DTF-based methods were developed. Ding et al. [29] proposed a short time directed transfer function (SDTF), in which the entire data were divided into short overlapping time intervals for computation of DTF measure on each interval. Ginter et al. [30] and Yi et al. [31] calculated SDTF to exhibit the casual relations of brain functional networks caused by motor imagery afterwards. Korzeniewska [32] first introduced a full frequency Directed Transfer Function (ffDTF) in which the normalization of DTF was performed over the full frequency band, then dDTF [32] was derived by multiplying ffDTF by partial coherence to only show direct connections while DTF and ffDTF reveal both direct and indirect connections. Billinger [33] showed the possibility of reliable classification of MI tasks through ffDTF and dDTF. Later in [34], dDTF was applied to feature extraction with power spectral density. Adaptive Directed Transfer Function (ADTF) was originally proposed by Wilke et al. [35] for abnormal physiological signals, such as those during epileptic seizures. The authors established time-varying coefficient matrices by Kalman filter algorithm, constructed MVAAR models and got time-varying brain functional networks. In the following years, ADTF was also extended to the construction of time-varying brain function networks of other EEG signals. Li et al. [15, 36] used ADTF to investigate the time-varying P300 network patterns and evaluate time-varying MI-EEG functional networks as well as observe MI information processing in different stages. Wang et al. [37] presented a novel wavelet-based directed transfer function (WDTF) method by combining the wavelet decomposition and the directed transfer function (DTF) algorithm for patient-specific seizure detection.

To sum up, DTF and its extended methods have successively been applied to construct brain functional networks and shown their effectiveness for different EEG signals. It is a potential problem that how to grasp the activated characteristics of MI-EEG signals to further improve DTF and find effective feature parameters to identify MI tasks. In order to fully represent the variation characteristics of MI-EEG in frequency domain, we will present a dynamic DTF, named as DDTF. The experiments are conducted based on publicly available MI-EEG data from BCI competition and real acquisition data from real-world, and the results suggest that this metric not only extracts effective information, but also achieves a high classification accuracy while only one or two order frequency domain model is used to select the best frequency band and calculate DDTF, demonstrating the adaptive and dynamic characteristics of MI-EEG.

The rest of the paper is organized as follows: in Sect. 2, the DDTF-based feature extraction method is described in detail. Experimental research is performed in Sect. 3. Section 4 provides the discussions, and conclusions are drawn in the final section.

## Methods

By adaptively selecting the frequency band and model order *m*, DTF is developed to generate DDTF, which is applied to construction and feature extraction of brain functional network, the main process is as follows.

### Preprocessing of MI-EEG signals

Suppose that $x_{0} \left( t \right) = \left[ {x_{01} \left( t \right),x_{02} \left( t \right), \ldots ,x_{{0N_{0} }} \left( t \right)} \right]^{{\text{T}}} \in R^{{N_{0} \times K_{0} }}$ represents the original MI-EEG signals, where $N_{0}$ and $K_{0}$ refer to the number of total channels and sample points, respectively.

- Step 1: Common average removal (CAR) filtering.
  ${\varvec{x}}_{0} \left( t \right)$ is spatially filtered with CAR filter at first and the filtered signal is expressed as
  1 $$x_{1} \left( t \right) = \left[ {x_{11} \left( t \right),x_{12} \left( t \right), \ldots ,x_{{1N_{0} }} \left( t \right)} \right]^{{\text{T}}} \in R^{{N_{0} \times K_{0} }} .$$
- Step 2: Optimal sample interval selection.
  The optimal sample interval [*a*, *b*] is selected with the most obvious event-related desynchronization (ERD)/event-related synchronization (ERS) physiological phenomenon and the MI-EEG signals in this time period are denoted as
  2 $$x_{2} \left( t \right) = \left[ {x_{21} \left( t \right),x_{22} \left( t \right), \ldots ,x_{{2N_{0} }} \left( t \right)} \right]^{{\text{T}}} \in R^{{N_{0} \times K}} ,$$
  where $$K = b - a + 1.$$
- Step 3: Bandpass filtering.
  The important information in EEG signals is often hidden in frequency with respect to allied cognitive tasks [38] and it is generally accepted that most of the motor imagery-related EEG signals are coded in the frequency band of 8–30 Hz [39]. What’s more, the $\beta { }$ band (13–30 Hz), with $\beta_{1}$ or lower $\beta$ sub-band (13–21 Hz) and $\beta_{2}$ or higher $\beta$ sub-band (21–30 Hz) included, has been reported to be good physiological predictors during motor imagery tasks compared to other frequency bands [40]. This information is significant, as it has been verified that the interdependency at different frequency ranges may own distinct physiological functions [41]. At this point, ${\varvec{x}}_{2} \left( t \right)$ is bandpass filtered to $\alpha$ band and $\beta$ band ($\beta_{1 }$ and $\beta_{2 } {\text{sub}}$ band if necessary) and the filtered signals are later described as
  3 $$\left\{ {\begin{array}{*{20}ll} {x_{2}^{\alpha } \left( t \right) = \left[ {x_{21}^{\alpha } \left( t \right),x_{22}^{\alpha } \left( t \right), \ldots ,x_{{2N_{0} }}^{\alpha } \left( t \right)} \right]^{T} \in R^{{N_{0} \times K}} } \\ {x_{2}^{\beta } \left( t \right) = \left[ {x_{21}^{\beta } \left( t \right),x_{22}^{\beta } \left( t \right), \ldots ,x_{{2N_{0} }}^{\beta } \left( t \right)} \right]^{T} \in R^{{N_{0} \times K}} } \\ {x_{2}^{{\beta_{1} }} \left( t \right) = \left[ {x_{21}^{{\beta_{1} }} \left( t \right),x_{22}^{{\beta_{1} }} \left( t \right), \ldots ,x_{{2N_{0} }}^{{\beta_{1} }} \left( t \right)} \right]^{T} \in R^{{N_{0} \times K}} } \\ {x_{2}^{{\beta_{2} }} \left( t \right) = \left[ {x_{21}^{{\beta_{2} }} \left( t \right),x_{22}^{{\beta_{2} }} \left( t \right), \ldots ,x_{{2N_{0} }}^{{\beta_{2} }} \left( t \right)} \right]^{T} \in R^{{N_{0} \times K}} } \\ \end{array} } \right..$$
- Step 4: Channel selection.
  Considering the computational complexity and feature information redundancy, $N$ channels are selected to cover as many brain regions as possible in the premise of guaranteeing their asymmetries, the signals after channel selection are represented as ${\varvec{x}}_{3}^{\alpha } \left( t \right) = \left[ {x_{31}^{\alpha } \left( t \right),x_{32}^{\alpha } \left( t \right), \ldots ,x_{3N}^{\alpha } \left( t \right)} \right] \in R^{N \times K} ,\;{\varvec{x}}_{3}^{\beta } \left( t \right) = \left[ {x_{31}^{\beta } \left( t \right),x_{32}^{\beta } \left( t \right), \ldots ,x_{3N}^{\beta } \left( t \right)} \right] \in R^{N \times K} ,$ ${\varvec{x}}_{3}^{{\beta_{1} }} \left( t \right) = \left[ {x_{31}^{{\beta_{1} }} \left( t \right),x_{32}^{{\beta_{1} }} \left( t \right), \ldots ,x_{3N}^{{\beta_{1} }} \left( t \right)} \right] \in R^{N \times K} , \;{\varvec{x}}_{3}^{{\beta_{2} }} \left( t \right) = \left[ {x_{31}^{{\beta_{2} }} \left( t \right)} \right.,$ $x_{32}^{{\beta_{2} }} \left( t \right), \ldots ,\left. {x_{3N}^{{\beta_{2} }} \left( t \right)} \right] \in R^{N \times K}$ and later rewritten as
  4 $$\left\{ {\begin{array}{*{20}ll} {{\varvec{x}}^{\alpha } \left( t \right) = \left[ {x_{1}^{\alpha } \left( t \right),x_{2}^{\alpha } \left( t \right), \ldots ,x_{N}^{\alpha } \left( t \right)} \right]^{{\text{T}}} \in R^{N \times K} } \\ {{\varvec{x}}^{\beta } \left( t \right) = \left[ {x_{1}^{\beta } \left( t \right),x_{2}^{\beta } \left( t \right), \ldots ,x_{N}^{\beta } \left( t \right)} \right]^{{\text{T}}} \in R^{N \times K} } \\ {{\varvec{x}}^{{\beta_{1} }} \left( t \right) = \left[ {x_{1}^{{\beta_{1} }} \left( t \right),x_{2}^{{\beta_{1} }} \left( t \right), \ldots ,x_{N}^{{\beta_{1} }} \left( t \right)} \right]^{{\text{T}}} \in R^{N \times K} } \\ { {\varvec{x}}^{{\beta_{2} }} \left( t \right) = \left[ {x_{1}^{{\beta_{2} }} \left( t \right),x_{2}^{{\beta_{2} }} \left( t \right), \ldots ,x_{N}^{{\beta_{2} }} \left( t \right)} \right]^{{\text{T}}} \in R^{N \times K} } \\ \end{array} } \right..$$

### The proposed dynamic DTF (DDTF)

In this subsection, DTF is improved, named as DDTF, in which the model order and frequency band are changing and adaptively selected by recognition rates. The detailed introduction takes $\alpha$ band for example.

- Step 1: Let us consider a multivariate autoregressive model (MVAR), the present state of MI-EEG signals can be approximated by a weighted sum of $p_{\alpha }$ previous values of all selected channels over $\alpha$ band, and a random noise series is also added:
  5 $${\varvec{x}}^{\alpha } \left( t \right) = \sum \limits_{r = 1}^{{p_{\alpha } }} {\varvec{A}}^{\alpha } \left( r \right){\varvec{x}}^{\alpha } \left( {t - r} \right) + {\varvec{e}}^{\alpha } \left( t \right),$$
  where $e^{\alpha } \left( t \right) = \left[ {e_{1}^{\alpha } \left( t \right),e_{2}^{\alpha } \left( t \right), \ldots ,e_{N}^{\alpha } \left( t \right)} \right]^{{\text{T}}}$ is the estimation error which is a multivariate uncorrelated white noise sequence with zero mean. $x^{\alpha } \left( {t - r} \right) = \left[ {x_{1}^{\alpha } \left( {t - r} \right),x_{2}^{\alpha } \left( {t - r} \right), \ldots ,x_{N}^{\alpha } \left( {t - r} \right)} \right]^{{\text{T}}}$ is an *N* *×* 1 vector of ${\varvec{x}}\left( t \right)$ at a time lag ‘*r*’. ${\varvec{A}}^{\alpha } \left( 1 \right),\;{\varvec{A}}^{\alpha } \left( 2 \right), \ldots ,{\varvec{A}}^{\alpha } \left( r \right)$ are the *N* *×* *N* matrices of coefficient matrices, e.g., ${\varvec{A}}^{\alpha } \left( r \right)$ is a matrix for describing the time-lagged influences of ${\varvec{x}}^{\alpha } \left( {t - r} \right)$ on ${\varvec{x}}^{\alpha } \left( t \right)$. Assume that each element of matrix ${\varvec{A}}^{\alpha } \left( r \right)$ is denoted as $a_{uv}^{\alpha }$, the coefficient matrix can be set as $${\mathbf{A}}^{\alpha } \left( r \right) = \left( {\begin{array}{*{20}ll} {a_{11}^{\alpha } \left( r \right)} & \cdots & {a_{1N}^{\alpha } \left( r \right)} \\ \vdots & \ddots & \vdots \\ {a_{N1}^{\alpha } \left( r \right)} & \cdots & {a_{NN}^{\alpha } \left( r \right)} \\ \end{array} } \right),$$ the off-diagonal parts $a_{uv}^{\alpha } \left( r \right),\;u \ne v$ quantify time-lagged influences between different channels of EEG signals $x_{u}^{\alpha } \left( t \right)$ and $x_{v}^{\alpha } \left( t \right)$. The model coefficients can be got iteratively so as to minimize the error between the actual (measured) and predicted values. The model order $p_{\alpha } ,$ which denotes the maximum time lag, is used to disclose the influences of past states on the current state. The optimal model order is determined by Schwarz Bayesian Criterion (SBC) [42] and is used for MVAR model fitting to MI-EEG data.
- Step 2: Once the MVAR model is fully constructed, the dynamic spectral analysis is performed based on Fourier transform. The ${\varvec{B}}^{{m_{\alpha } }} \left( f \right)$, which has a varying order $m_{\alpha } ,$ is defined as follows:
  6 $${\varvec{B}}^{{m_{\alpha } }} \left( f \right) = - \sum \limits_{r = 0}^{{m_{\alpha } }} {\varvec{A}}^{\alpha } \left( r \right)e^{ - j2\pi fr\Delta t} ,m_{\alpha } = 1,2, \ldots ,p_{\alpha } ,$$
  where ${\varvec{B}}^{0} \left( f \right) = - {\mathbf{I}}$ (**I** being the identity matrix).${ }\Delta t$ is the temporal interval between two samples, $j$ represents the imagery unit, $f$ denotes frequency, and we have
  7 $${\varvec{B}}^{{m_{\alpha } }} \left( f \right){\varvec{X}}^{\alpha } \left( f \right) = {\varvec{E}}^{\alpha } \left( f \right),$$
  where ${\varvec{X}}^{\alpha } \left( f \right)$ and ${\varvec{E}}^{\alpha } \left( f \right)$ are the transforms of ${\varvec{x}}^{\alpha } \left( t \right)$ and ${\varvec{e}}^{\alpha } \left( t \right)$, respectively.
- Step 3: Equation (7) can be rewritten as follows:
  8 $${\varvec{X}}^{\alpha } \left( f \right) = \left[ {{\varvec{B}}^{{m_{\alpha } }} \left( f \right)} \right]^{ - 1} {\varvec{E}}^{\alpha } \left( f \right) = {\varvec{H}}^{{m_{\alpha } }} \left( f \right){\varvec{E}}^{\alpha } \left( f \right).$$
  Here ${\varvec{H}}^{{m_{\alpha } }} \left( f \right)$ is called the transfer matrix, and based on it we define the DDTF from channel *s* to channel *l* at frequency *f* as follows:
  9 $$\left[ {\theta_{ls}^{{m_{\alpha } }} \left( f \right)} \right]^{2} = \left| {H_{ls}^{{m_{\alpha } }} \left( f \right)} \right|^{2} ,$$
  where $H_{ls}^{{m_{\alpha } }} \left( f \right)$ is the element in the *l*th row of column *s* of transfer matrix ${\varvec{H}}^{{m_{\alpha } }} \left( f \right)$. In addition, a normalization procedure is further executed when $\left[ {\theta_{ls}^{{m_{\alpha } }} \left( f \right)} \right]^{2}$ is divided by the quadratic sum of all elements in the *l*th row as follows:
  10 $$\left[ {\gamma_{ls}^{{m_{\alpha } }} \left( f \right)} \right]^{2} = \frac{{\left[ {\theta_{ls}^{{m_{\alpha } }} \left( f \right)} \right]^{2} }}{{ \sum \nolimits_{q = 1}^{N} \left[ {\theta_{lq}^{{m_{\alpha } }} \left( f \right)} \right]^{2} }},$$
  which describes the ratio of influence from channel *s* to channel *l* with respect to the joint influence from all the other channels to channel *l* and has a value between 0 to 1. Value close to 1 means that the channel *s* has great impact on the channel *l* while value close to 0 shows that the channel *s* makes little contribution to channel *l*.
- Step 4: To get the mean value of Eq. (10) under different frequencies, the accumulation over $\alpha$ band is applied:
  11 $$\overline{{\Upsilon_{ls}^{{m_{\alpha } }} }} = \frac{{ \sum \nolimits_{{f = f_{1} }}^{{f_{2} }} \left[ {\gamma_{ls}^{{m_{\alpha } }} \left( f \right)} \right]^{2} }}{{f_{2} - f_{1} + 1}}.$$
  Here $$f_{1} f_{1} ,$$ $$f_{2}$$ equal to 8 Hz and 13 Hz (i.e., the lower and upper bound of $$\alpha { }$$ band), respectively. Perform the same steps for $${\varvec{x}}^{\beta } \left( t \right),$$$${\varvec{x}}^{{\beta_{1} }} \left( t \right),$$ $${\varvec{x}}^{{\beta_{2} }} \left( t \right)$$ and we get
  12 $$\left\{ {\begin{array}{*{20}ll} {\overline{{\Upsilon_{ls}^{{m_{\beta } }} }} = \frac{{ \sum \nolimits_{{f = f_{2} }}^{{f_{4} }} \left[ {\gamma_{ls}^{{m_{\beta } }} \left( f \right)} \right]^{2} }}{{f_{4} - f_{2} + 1}}} \\ {\overline{{ \Upsilon_{ls}^{{m_{{\beta_{1} }} }} }} = \frac{{ \sum \nolimits_{{f = f_{2} }}^{{f_{3} }} \left[ {\gamma_{ls}^{{m_{{\beta_{1} }} }} \left( f \right)} \right]^{2} }}{{f_{3} - f_{2} + 1}}} \\ {\overline{{\Upsilon_{ls}^{{m_{{\beta_{2} }} }} }} = \frac{{ \sum \nolimits_{{f = f_{3} }}^{{f_{4} }} \left[ {\gamma_{ls}^{{m_{{\beta_{2} }} }} \left( f \right)} \right]^{2} }}{{f_{4} - f_{3} + 1}}} \\ \end{array} } \right.,$$
  where $f_{3} ,$ $f_{4}$ are 21 Hz and 30 Hz, respectively.

### Brain functional network construction based on DDTF

$\overline{{\Upsilon_{ls}^{{m_{\alpha } }} }}$ reveals the direction and strength between two channels in the total $\alpha$ band. For example, $\overline{{\Upsilon_{12}^{{m_{\alpha } }} }}$ represents the connection intensity from channel 2 to channel 1 and vice versa. The brain functional network of $\alpha$ band then can be constructed with EEG electrodes as nodes and $\overline{{{{\varvec{\Upsilon}}}^{{m_{\alpha } }} }} ,$ the adjacency matrix (AM), as links between channels. Similarly, the brain functional networks of $\beta ,$ ${ }\beta_{1}$ and $\beta_{2}$ bands can also be constructed separately.

### Definition of feature parameters

Feature parameters can be obtained according to the brain functional networks and adjacency matrices. Given channel *g* (*g* = 1, 2,$$\ldots$$, *N*) for example, the *g*th row of feature matrix $$\overline{{{{\varvec{\Upsilon}}}^{{m_{\alpha } }} }} ,$$$$\overline{{{{\varvec{\Upsilon}}}^{{m_{\beta } }} }} ,\;\overline{{{{\varvec{\Upsilon}}}^{{m_{{\beta_{1} }} }} }} \;{\text{and}}\; \overline{{{{\varvec{\Upsilon}}}^{{m_{{\beta_{2} }} }} }}$$ are summed to acquire the inflow information to channel *g* for $$\alpha ,\; \beta ,$$$${ }\beta_{1}$$ and $$\beta_{2}$$ bands, respectively:

13 $$\left\{ {\begin{array}{*{20}ll} {{\text{IN}}^{{m_{\alpha } }} \left( g \right) = \sum \limits_{s = 1}^{N} \overline{{\Upsilon_{gs}^{{m_{\alpha } }} }} } \\ {{\text{IN}}^{{m_{\beta } }} \left( g \right) = \sum \limits_{s = 1}^{N} \overline{{\Upsilon_{gs}^{{m_{\beta } }} }} } \\ {{\text{IN}}^{{m_{{\beta_{1} }} }} \left( g \right) = \sum \limits_{s = 1}^{N} \overline{{\Upsilon_{gs}^{{m_{{\beta_{1} }} }} }} } \\ {{\text{IN}}^{{m_{{\beta_{2} }} }} \left( g \right) = \sum \limits_{s = 1}^{N} \overline{{\Upsilon_{gs}^{{m_{{\beta_{2} }} }} }} } \\ \end{array} } \right..$$

As a logical sequence, the outflow from channel *g* is obtained by summing the *g*th column of adjacency matrix for each band as follows:

14 $$\left\{{\begin{array}{*{20}ll} {{\text{OUT}}^{{m_{\alpha } }} \left( g \right) = \sum \limits_{l = 1}^{N} \overline{{\Upsilon_{lg}^{{m_{\alpha } }} }} } \\ {{\text{OUT}}^{{m_{\beta } }} \left( g \right) = \sum \limits_{l = 1}^{N} \overline{{\Upsilon_{lg}^{{m_{\beta } }} }} } \\ {{\text{OUT}}^{{m_{{\beta_{1} }} }} \left( g \right) = \sum \limits_{l = 1}^{N} \overline{{\Upsilon_{lg}^{{m_{{\beta_{1} }} }} }} } \\ {{\text{OUT}}^{{m_{{\beta_{2} }} }} \left( g \right) = \sum \limits_{l = 1}^{N} \overline{{\Upsilon_{lg}^{{m_{{\beta_{2} }} }} }} } \\ \end{array} } \right..$$

Inflow characterizes the total information received by a particular destination channel *g* from other channels while outflow describes gross messages transmitted from the given source node *g* to the rest of network. Both explore the information interaction processes between specific areas of the brain and other regions.

Furthermore, the inflow and outflow are combined to define information flow (similarly, we take the channel *g* as an example):

15 $${\text{IF}}\left\{ {\begin{array}{*{20}ll} {{\text{IF}}^{{m_{\alpha } }} \left( g \right) = \frac{{{\text{OUT}}^{{m_{\alpha } }} \left( g \right)}}{{{\text{IN}}^{{m_{\alpha } }} \left( g \right)}} } \\ {{\text{IF}}^{{m_{\beta } }} \left( g \right) = \frac{{{\text{OUT}}^{{m_{\beta } }} \left( g \right)}}{{{\text{IN}}^{{m_{\beta } }} \left( g \right)}}} \\ {{\text{IF}}^{{m_{{\beta_{1} }} }} \left( g \right) = \frac{{{\text{OUT}}^{{m_{{\beta_{1} }} }} \left( g \right)}}{{{\text{IN}}^{{m_{{\beta_{1} }} }} \left( g \right)}} } \\ {\text{IF}^{{m_{{\beta_{2} }} }} \left( g \right) = \frac{{{\text{OUT}}^{{m_{{\beta_{2} }} }} \left( g \right)}}{{{\text{IN}}^{{m_{{\beta_{2} }} }} \left( g \right)}} } \\ \end{array} .} \right.$$

Information flow indicates the role of channel *g* playing in the process of information transmission. The larger information flow is, the greater contribution *g* has to other channels. Conversely, it means that there is barely no or very little info from channel *g* when the value is approaching to 0.

### Construction of a feature vector

Let us take $\alpha$ band for example, there is an ${\text{OUT}}^{{m_{\alpha } }} \left( g \right)$ and ${\text{IF}}^{{m_{\alpha } }} \left( g \right)$ for channel *g*, ${\varvec{OUT}}^{{m_{\alpha } }}$ and ${\varvec{IF}}^{{m_{\alpha } }}$ can be obtained when the features related to all the *N* channels are assembled, namely, ${\varvec{OUT}}^{{m_{\alpha } }} = \left[ {{\text{OUT}}^{{m_{\alpha } }} \left( 1 \right),{\text{OUT}}^{{m_{\alpha } }} \left( 2 \right), \ldots ,{\text{OUT}}^{{m_{\alpha } }} \left( g \right), \ldots ,{\text{OUT}}^{{m_{\alpha } }} \left( N \right)} \right] \in R^{1 \times N} ,$ ${\varvec{IF}}^{{m_{\alpha } }} = \left[ {{\text{IF}}^{{m_{\alpha } }} \left( {1} \right),{\text{IF}}^{{m_{\alpha } }} \left( 2 \right), \ldots ,{\text{IF}}^{{m_{\alpha } }} \left( g \right), \ldots ,{\text{IF}}^{{m_{\alpha } }} (N)} \right] \in R^{1 \times N}$, where *N* means the number of selected channels. They are fused in serial to form the network feature vector of *α* band, namely ${\varvec{F}}^{{m_{\alpha } }}$:

16 $${\varvec{F}}^{{m_{\alpha } }} = \left[ {{\varvec{IF}}^{{m_{\alpha } }} ,{\varvec{OUT}}^{{m_{\alpha } }} } \right] \in R^{1 \times 2N} .$$

Analogously, feature vectors of $\beta ,$ ${ }\beta_{1}$ or $\beta_{2}$ band can be acquired as follows:

17 $$\left\{ {\begin{array}{*{20}ll} {{\varvec{F}}^{{m_{\beta } }} = \left[ {{\varvec{IF}}^{{m_{\beta } }} ,{\varvec{OUT}}^{{m_{\beta } }} } \right] \in R^{1 \times 2N} } \\ {{\varvec{F}}^{{m_{{\beta_{1} }} }} = \left[ {{\varvec{IF}}^{{m_{{\beta_{1} }} }} ,{\varvec{OUT}}^{{m_{{\beta_{1} }} }} } \right] \in R^{1 \times 2N} } \\ {{\varvec{F}}^{{m_{{\beta_{2} }} }} = \left[ {{\varvec{IF}}^{{m_{{\beta_{2} }} }} ,{\varvec{OUT}}^{{m_{{\beta_{2} }} }} } \right] \in R^{1 \times 2N} } \\ \end{array} } \right..$$

After this process, feature vectors at a characteristic frequency for those directed interconnections are input to SVM classifier to discriminate different motor imagery tasks.

### Feature evaluation by SVM

Given each $m_{\alpha }$, we have the corresponding features ${\varvec{F}}^{{m_{\alpha } }}$ and accuracies ${\text{Acc}}^{{m_{\alpha } }}$. Similarly, ${\varvec{F}}^{{m_{\beta } }}$ and ${\text{Acc}}^{{m_{\beta } }}$ can be obtained when MI-EEG signals are filtered to $\beta$ band. As is said before, motor imagery response frequency bands are not consistent for each individual subject. Therefore, it is of vital importance to find subject-specific frequency band, and accuracies in $\alpha$ band and $\beta$ band are compared. Suppose ${\text{Acc}}^{{m_{\alpha } }}$ are higher than ${\text{Acc}}^{{m_{\beta } }}$, it means $\alpha$ band is the final frequency band we are seeking for. Instead, the best classification accuracy is hidden in $\beta$ band. Filter MI-EEG signals to $\beta_{1 }$(13–21 Hz) and $\beta_{2}$ band (21–30 Hz), repeat the above operations and the best accuracy as well as frequency band can ultimately be acquired.

The DDTF algorithm used for brain functional network-based feature extraction is as follows:

**Table:** 

**Input:** MI-EEG signals ${\varvec{x}}_{0} \left( t \right)$
Preprocessing of MI-EEG signals
Step 1. Common average removal (CAR) filtering
Step 2. Optimal sample interval selection
Step 3. Bandpass filter to ${ }\alpha$ (8–13 Hz) and $\beta$ band (13–30 Hz)
Step 4. Channel selection, get ${\varvec{x}}^{\alpha } \left( t \right)$ and ${\varvec{x}}^{\beta } \left( t \right)$
Adjacency matrix calculation based on DDTF
Step 1. MVAR model fitting to ${\varvec{x}}^{\alpha } \left( t \right)$ and ${\varvec{x}}^{\beta } \left( t \right)$, respectively
Step 2. Calculate ${\varvec{B}}^{{m_{\alpha } }} \left( f \right)$ and ${\varvec{B}}^{{m_{\beta } }} \left( f \right)$ using Eq. (6)
Step 3. Calculate DDTF and adjacency matrix of $\left[ {\theta_{ls}^{{m_{\alpha } }} \left( f \right)} \right]^{2}$, $\left[ {\theta_{ls}^{{m_{\beta } }} \left( f \right)} \right]^{2}$ and $\overline{{{{\varvec{\Upsilon}}}^{{m_{\alpha } }} }}$, $\overline{{{{\varvec{\Upsilon}}}^{{m_{\beta } }} }}$ using Eqs. (8) to (12)
Definition of feature parameters
Step 1. Extract the features of inflow ${\text{IN}}^{{m_{\alpha } }} \left( g \right)$ and ${\text{IN}}^{{m_{\beta } }} \left( g \right)$ by Eq. (13)
Step 2. Extract the features of outflow ${\text{OUT}}^{{m_{\alpha } }} \left( g \right)$ and ${\text{OUT}}^{{m_{\beta } }} \left( g \right)$ by Eq. (14)
Step 3. Extract the features of information flow ${\text{IF}}^{{m_{\alpha } }} \left( g \right)$ and ${\text{IF}}^{{m_{\beta } }} \left( g \right)$ by Eq. (15)
Construction of a feature vector
Obtain ${\varvec{OUT}}^{{m_{\alpha } }} ,$ ${\varvec{IF}}^{{m_{\alpha } }}$ and ${\varvec{OUT}}^{{m_{\beta } }} ,$ ${\varvec{IF}}^{{m_{\beta } }} ,$ yield feature vector ${\varvec{F}}^{{m_{\alpha } }}$ and ${\varvec{F}}^{{m_{\beta } }}$
Feature evaluation by SVM
Compare $${\text{Acc}}^{{m_{\alpha } }}$$ with $${\text{Acc}}^{{m_{\beta } }}$$
**If** ${\text{Acc}}^{{m_{\alpha } }}$ are higher than ${\text{Acc}}^{{m_{\beta } }}$
**Output:** the best accuracy of ${\upalpha }$ band, i.e.,$\alpha^{ba}$
**Else If** ${\text{Acc}}^{{m_{\alpha } }}$ are lower than ${\text{Acc}}^{{m_{\beta } }}$
Preprocessing of MI-EEG signals
Step 1. Common average removal (CAR) filtering
Step 2. Optimal sample interval selection
Step 3. Bandpass filter to ${ }\beta_{1}$ (13–21 Hz) and $\beta_{2}$ band (21–30 Hz)
Step 4. Channel selection, get ${\varvec{x}}^{{\beta_{1} }} \left( t \right)$ and ${\varvec{x}}^{{\beta_{2} }} \left( t \right)$
Adjacency matrix calculation based on DDTF
Step 1. MVAR model fitting to ${\varvec{x}}^{{\beta_{1} }} \left( t \right)$ and ${\varvec{x}}^{{\beta_{2} }} \left( t \right)$, respectively
Step 2. Calculate ${\varvec{B}}^{{m_{{\beta_{1} }} }} \left( f \right)$ and ${\varvec{B}}^{{m_{{\beta_{2} }} }} \left( f \right)$ using Eq. (6)
Step 3. Calculate DDTF and adjacency matrix of $\left[ {\theta_{ls}^{{m_{{\beta_{1} }} }} \left( f \right)} \right]^{2}$, $\left[ {\theta_{ls}^{{m_{{\beta_{2} }} }} \left( f \right)} \right]^{2}$ and $\overline{{{{\varvec{\Upsilon}}}^{{m_{{\beta_{1} }} }} }}$, $\overline{{{{\varvec{\Upsilon}}}^{{m_{{\beta_{2} }} }} }}$ using Eqs. (8) to (12)
Definition of feature parameters
Step 1. Extract the features of inflow ${\text{IN}}^{{m_{{\beta_{1} }} }} \left( g \right)$ and ${\text{IN}}^{{m_{{\beta_{2} }} }} \left( g \right)$ by Eq. (13)
Step 2. Extract the features of outflow ${\text{OUT}}^{{m_{{\beta_{1} }} }} \left( g \right)$ and ${\text{OUT}}^{{m_{{\beta_{2} }} }} \left( g \right)$ by Eq. (14)
Step 3. Extract the features of information flow ${\text{IF}}^{{m_{{\beta_{1} }} }} \left( g \right)$ and ${\text{IF}}^{{m_{{\beta_{2} }} }} \left( g \right)$ by Eq. (15)
Construction of a feature vector
Obtain ${\varvec{OUT}}^{{m_{{\beta_{1} }} }} ,{\varvec{IF}}^{{m_{{\beta_{1} }} }}$ and ${\varvec{OUT}}^{{m_{{\beta_{2} }} }} ,{\varvec{IF}}^{{m_{{\beta_{2} }} }} ,$ yield feature vector ${\varvec{F}}^{{m_{{\beta_{1} }} }}$ and ${\varvec{F}}^{{m_{{\beta_{2} }} }}$
Feature evaluation by SVM
Compare $${\text{Acc}}^{{m_{\beta } }} ,$$ $${\text{Acc}}^{{m_{{\beta_{1} }} }}$$ and $${\text{Acc}}^{{m_{{\beta_{2} }} }}$$
**Output:** the optimal frequency band and the best accuracy

## Experimental research

### Data description and preprocessing

The proposed method was first examined in detail based on a publicly available motor imagery dataset, which was provided by BCI Lab, Graz University of Technology. The datasets generated and analyzed during current study is available in the BCI Competition III Dataset IIIa repository, http://www.bbci.de/competition/iii [43]. After that, it was applied to a second motor imagery dataset which was acquired from a real-world experiment. The dataset is available from the corresponding author on reasonable request. For easy of description, the referenced datasets were renamed as Dataset A and Dataset B below.

#### Dataset A: BCI competition III Dataset IIIa

**Fig. 1:**
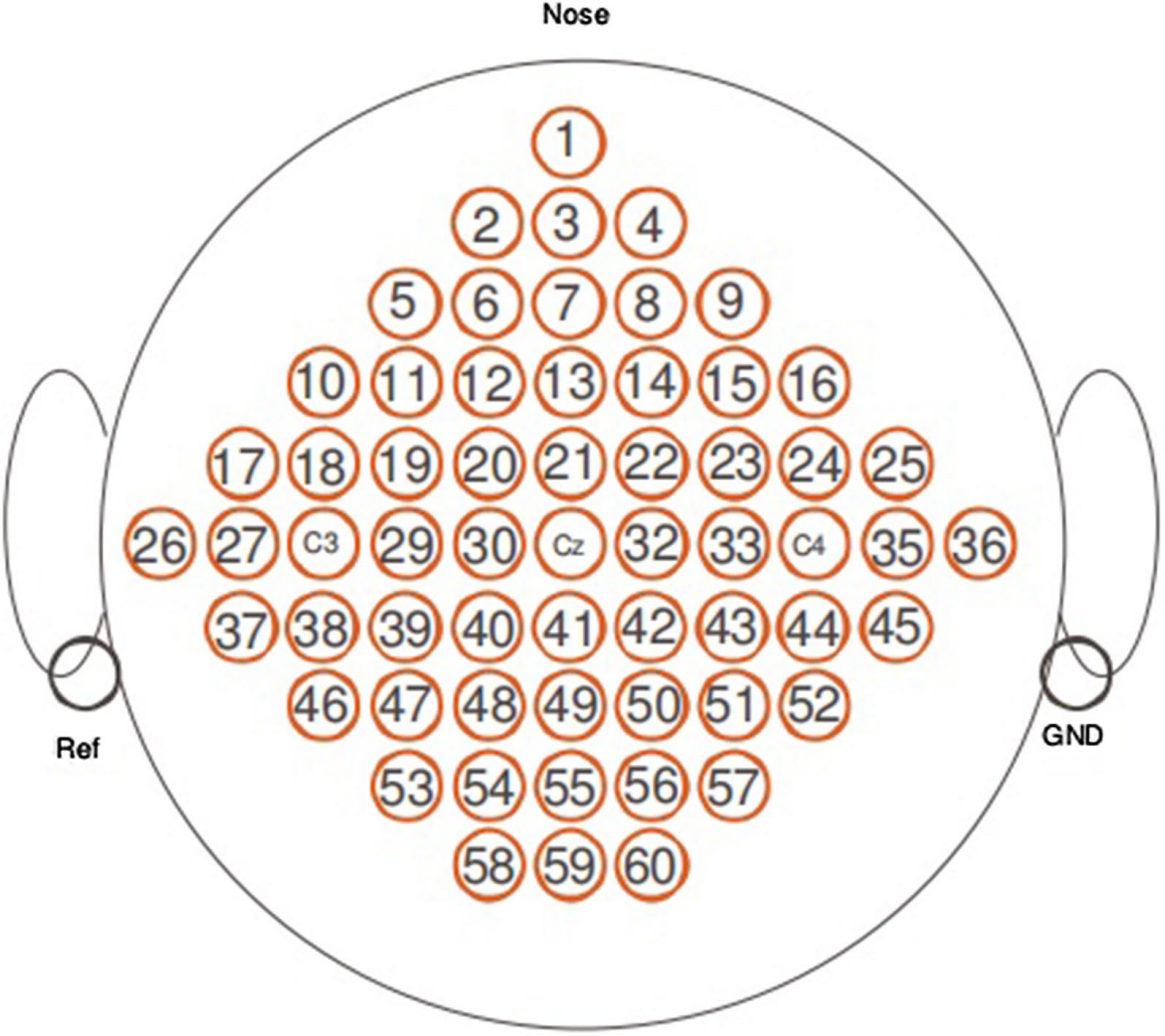
Electrode positions of Dataset A

**Fig. 2:**
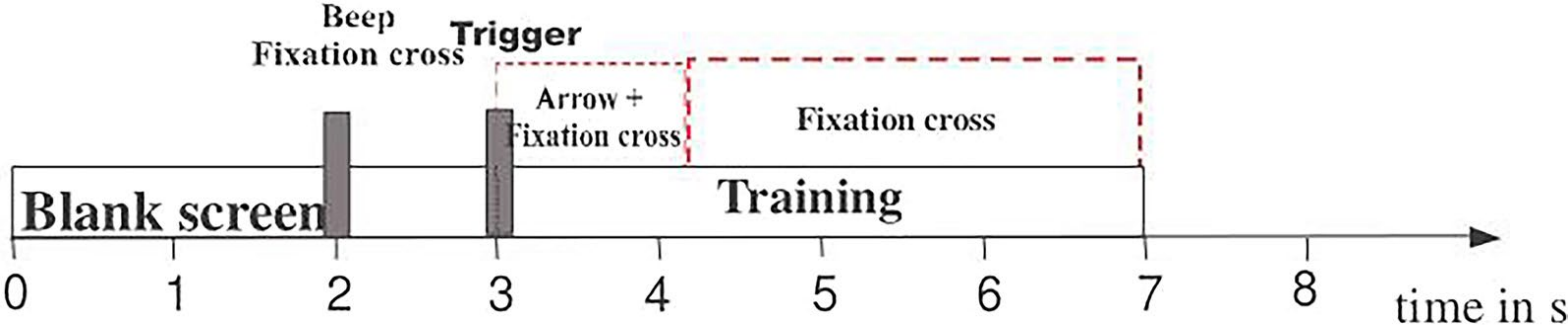
Timing diagram of Dataset A

The public EEG dataset considered originally consists of 60 EEG recordings referenced to the left mastoid and with the right mastoid serving as ground, the electrode position distribution is shown according to the scheme in Fig. 1. EEG was sampled at 250 Hz, it was online filtered by a bandpass filter between 1 and 50 Hz to remove artifacts. Dataset consists of three subjects performing left hand and right hand motor imagery tasks. A training set and a testing set are available for each subject. Both sets contain 45 trials per class for subject 1 (labeled as ‘k3b’), and 30 trials per class for subjects 2 and 3 (labeled as ‘k6b’ and ‘l1b’). The subjects sat in a comfortable chair with armrests and were instructed to perform a specific motor imagery task once the relevant cue was shown on a screen, until a fixation cross disappeared. Each trial lasted 8 s. After a trial began, the first 2 s was quiet, at *t* = 2 s an acoustic stimulus indicated the beginning of the trial, and a cross “+”was displayed; then from *t* = 3 s an arrow to the left or right was displayed for 1 s; at the same time the subject was asked to imagine left hand or right hand movement, respectively, until the cross disappeared at *t* = 7 s. The subject took a break and then conducted the next experiment. The MI-EEG collection timing scheme is shown in Fig. 2.

In the preprocessing stage, common average reference method was firstly applied to spatial filtering. Each trial extracted from the time segment between 3.5 and 7 s and was spectral filtered in $\alpha$ band (8–13 Hz) and $\beta$ band (13–30 Hz) (If necessary, EEG signals would be filtered to ${ }\beta_{{1{ }}}$(13–21 Hz) and ${ }\beta_{{2{ }}} { }$(21–30 Hz) band as well). In order to reduce the computational complexity, 34 channels of MI-EEG were selected symmetrically to carry out the experiment, and the selected electrodes cover as many brain regions as possible. The chosen sensor numbers are 1, 2, 3, 4, 5, 7, 9, 11, 13, 15, 17, 19, 21, 23, 25, 27, 29, 31, 33, 35, 37, 39, 41, 43, 45, 47, 49, 51, 53, 55, 57, 58, 59 and 60, respectively. Renumber these 34 channel numbers as 1–34 for subsequent experimental research.

#### Dataset B: real acquisition data

**Fig. 3:**
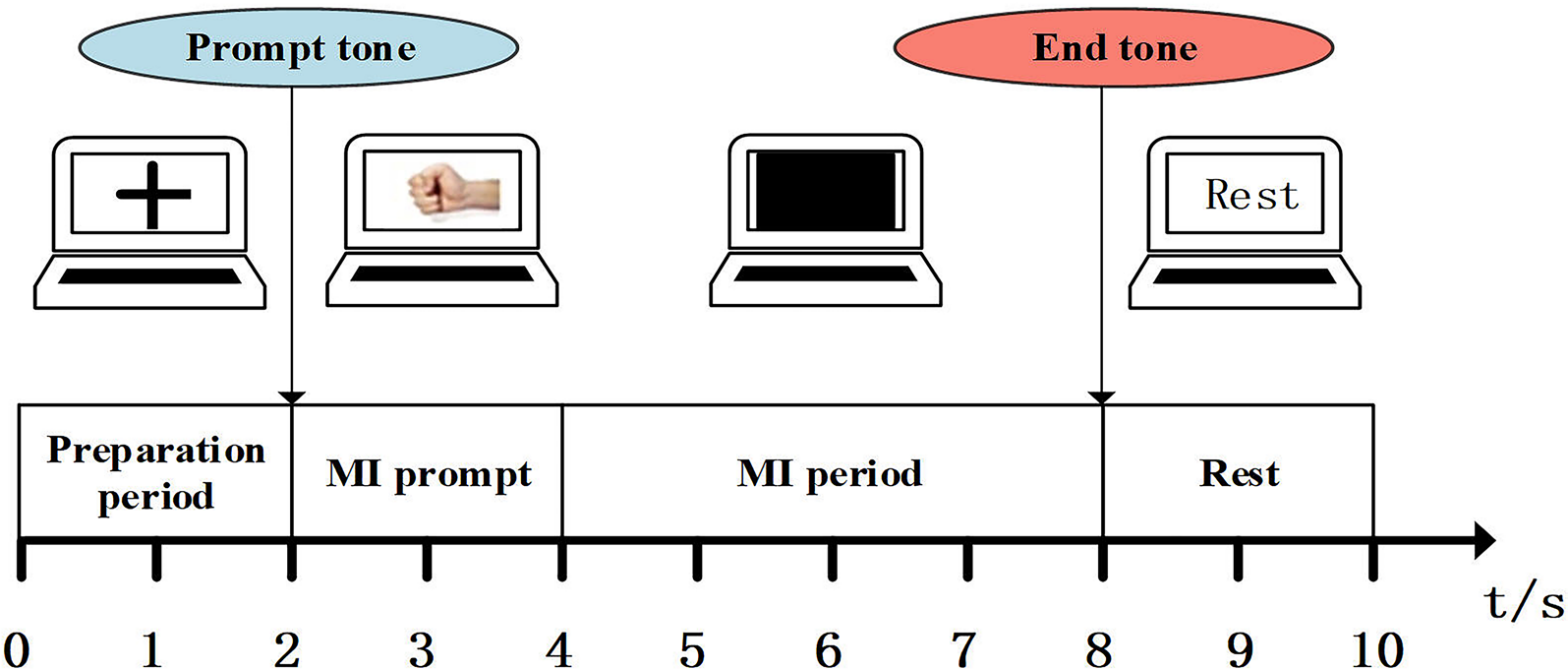
Timing diagram of Dataset B

**Fig. 4:**
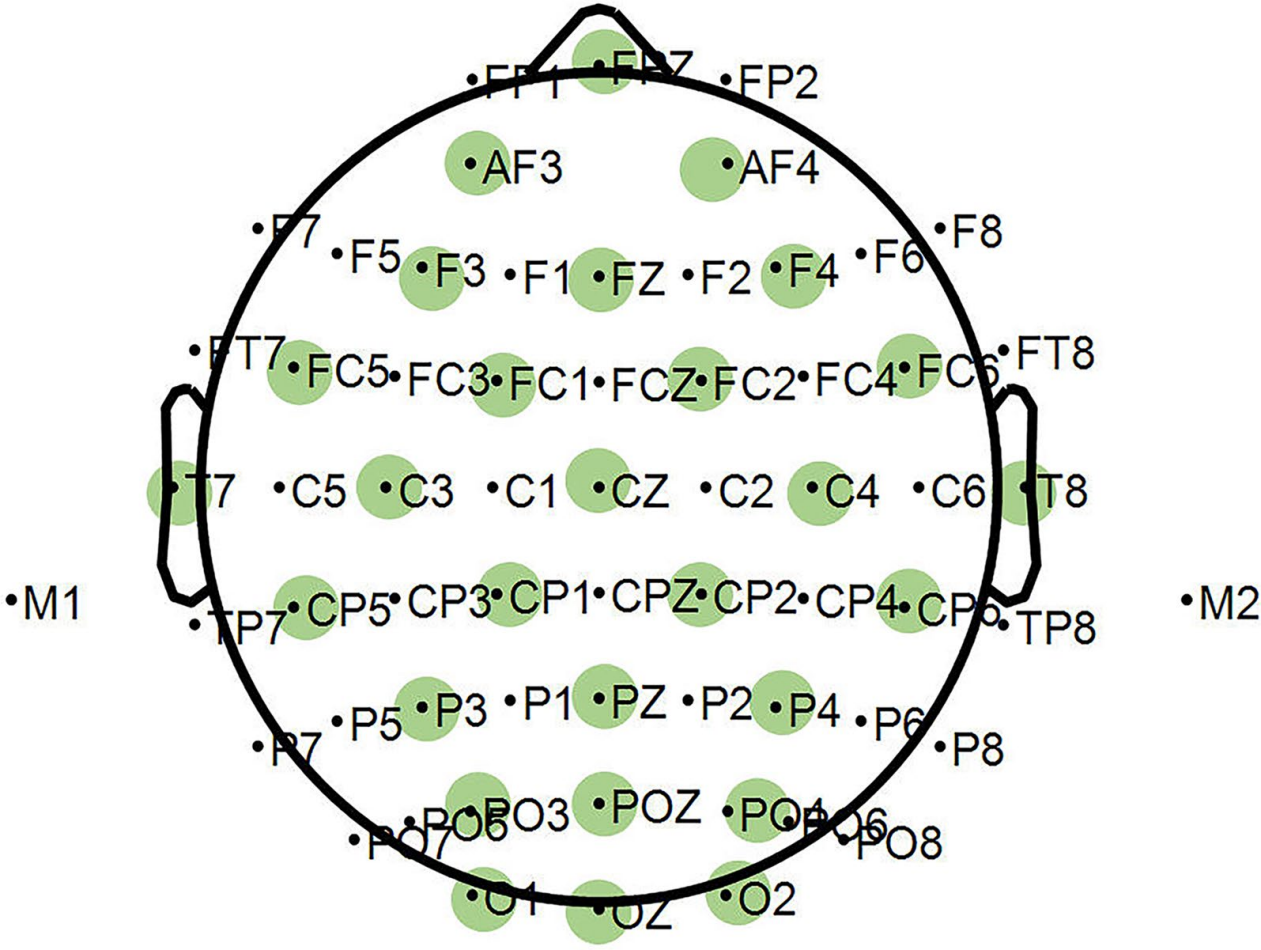
The selected channels of Dataset B

The real MI-EEG data were acquired by Neuroscan EEG signal recording and analysis system. EEGs were collected using 64 Ag/AgCl electrodes placed according to the international 10–20 system. The subjects are 9 healthy graduate students aged between 23 and 26 (marked as S1–S9, respectively), including two females and seven males. All of them are right-handed and without cognitive impairment. The MI tasks included imagining right-hand pronation and supination, and the sampling frequency was 1000 Hz. In order to ensure the quality of MI-EEG signals, the acquisition experiment of each subject was divided into 5 runs, one run consists of 20 trials (10 for each of two possible classes), i.e., 50 trials per class for each subject. Each subject should be trained in 2 runs of MI tasks before formal acquisition experiment to guarantee he/she was familiar with the experimental process. Each trial lasted 8 s, and the timing diagram is shown in Fig. 3. The first 2 s was preparation period, the “+” cursor appeared on the screen; at *t* = 2 s the motor imagery prompt tone appeared, and the prompt video was displayed on the screen for 2 s, i.e., imagining the right-hand pronation or supination; the subject performed the corresponding MI task according to the video prompt at 4–8 s; the end sound of MI task appeared on 8 s, the subject took a rest and then proceeded to the next experiment. Thereafter, the original collected MI-EEG signals were down-sampled to 250 Hz and the ocular artifacts were removed. Then, a CAR spatial filter was used for baseline correction. The 4.5–7.5 s MI-EEG was further intercepted and filtered to different frequency bands. Meanwhile, 28 channels covered by green circles in Fig. 4 are selected to reduce information redundancy and they are channels FPZ, AF3, AF4, F3, FZ, F4, FC5, FC1, FC2, FC6, T7, C3, CZ, C4, T8, CP5, CP1, CP2, CP6, P3, PZ, P4, PO3, POZ, PO4, O1, OZ and O2.

### Experimental results on Dataset A

#### Adaptive selection of the best frequency band and *m* value for each subject

**Fig. 5:**
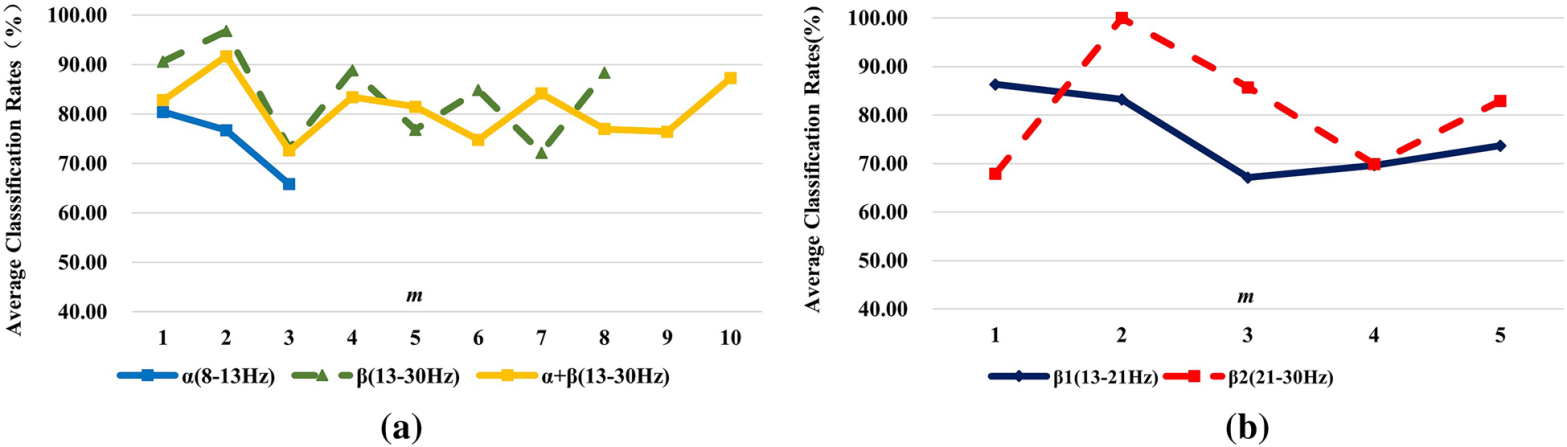
Effects of different frequency bands and *m* values on the classification results for subject ‘k3b’ **a** $\alpha$, $\beta$ and $\alpha$ + $\beta$ band; **b** $\beta_{1}$ and $\beta_{2}$ band

For subject ‘k3b’, MVAR were fitted to the preprocessed MI-EEG data (both $\alpha$ and $\beta$ band) and the model order of $\alpha$ and $\beta$ band were $p_{\alpha }$ = 3 and $p_{\beta }$ = 8, respectively. As is known to us all, the wider frequency band the signal, the more complex the signal is. Obviously, a higher model order is needed to match and describe the signal. Furthermore, ${\varvec{B}}^{{m_{\alpha } }} \left( f \right)$ and ${\mathbf{B}}^{{m_{\beta } }} \left( f \right)$ were calculated according to Eq. (6). Following the steps in Sect. 2, features ${\varvec{F}}^{{m_{\alpha } }}$ and ${\varvec{F}}^{{m_{\beta } }}$ were finally obtained and then assessed by SVM. For comparative research, experiments were also carried on MI-EEG signals filtered to 8–30 Hz with the model order of 10. The 10 × 10-fold cross-validation (CV) methodology was used to eliminate the contingency in feature extraction of MI-EEG signals as well as increase the reliability of experimental results, i.e., it uses nine observations to train the classifiers and the remaining one for testing the model. The final classification accuracy is the average of 10 runs [44], as is shown in Fig. 5a and *m* refers to the varying model order in subsequent sections.

It can be clearly seen from Fig. 5a that the highest recognition rate without frequency band selection, i.e., 91.67%, is lower than 96.78%, which is the best accuracy after band selection. In addition, classification accuracies of $\beta$ band are always higher than those of $\alpha$ band. Considering $\beta$ band is broader and may contain redundant information, it is refined to find the most active band for recognition. Under this consideration, we separated $\beta$ band into 2 sub-bands, i.e., $\beta_{1}$ band or lower $\beta$ band (13–21 Hz) and $\beta_{2}$ band or higher $\beta$ band (21–30 Hz). The same experiments were performed on MI-EEG signals in the two sub-bands, as is displayed in Algorithm. The model orders of $\beta_{1}$ and $\beta_{2}$ band were 5 and final recognition rates are exhibited in Fig. 5b for better observations. In Fig. 5b, the signals in $\beta_{2}$ band possess higher accuracies than those in $\beta_{1 }$ band except *m* is 1. Unbelievably and reasonably, the recognition rate reaches 100% when $m$ is set to 2 in $\beta_{2}$ band.

**Fig. 6:**
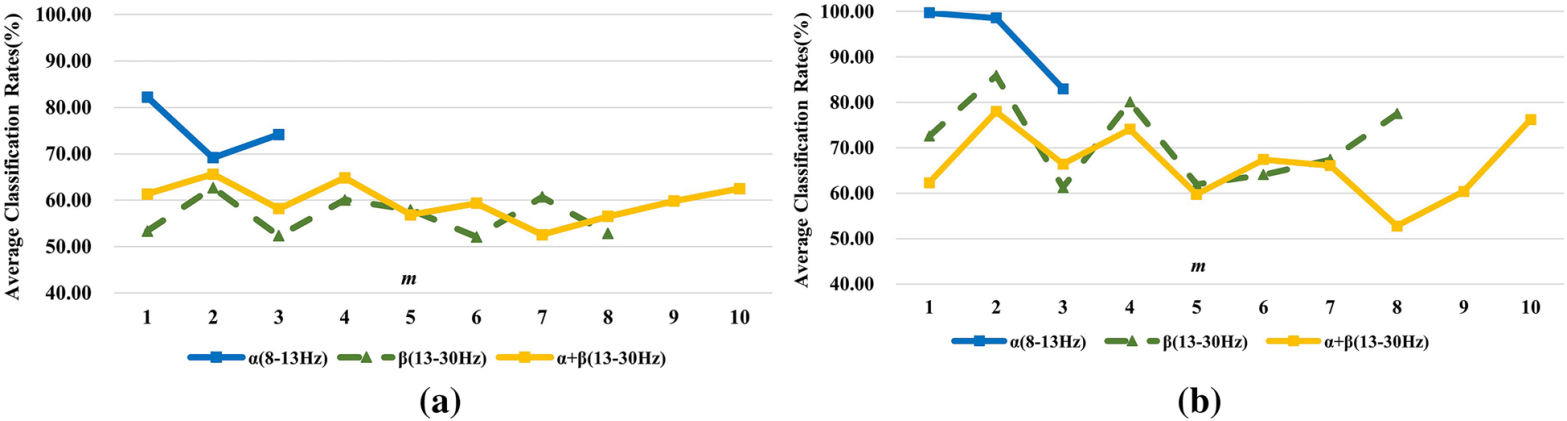
Effects of different frequency bands and *m* values on the classification results for subject **a** ‘k6b’ and **b** ‘l1b’

The same operations were carried on for subjects ‘k6b’ and ‘l1b’, the average recognition rates with 10 × 10-fold CV are demonstrated in Fig. 6a for ‘k6b’ and Fig. 6b for ‘l1b’, respectively.

Different from subject ‘k3b’, subjects ‘k6b’ and ‘l1b’ show better separability in $\alpha { }$ band comparing to $\beta { }$ band under left/right hand motor imagery tasks. Nevertheless, subject ‘l1b’ performs better than ‘k6b’ and gets higher accuracies. This phenomenon perfectly tallies with subject-based characteristic of MI-EEG signals. Affected by internal and external environments, subjects show great diversities. Even for the same subject, the recognition rates vary a lot in various frequency bands, as shown in Fig. 5a, b. Moreover, classification results of MI-EEG signals filtered to 8–30 Hz are worse than those under adaptive frequency band selection ($\alpha { }$ band) for subjects ‘k6b’ and ‘l1b’. All the above reveals that dividing EEG signals into varying frequency bands can improve the classification performances.

Similarly, recognition rates get largely promoted when *m* is set to 1 or 2 instead of the original model order *p* despite in either frequency band for all the subjects. This is because MI-EEG signals are non-stationary in frequency domain, the information of previous 1 or 2 moment is much more related to the current state and thus is more beneficial for recognition than all the information is considered. According to Figs. 5 and 6, the MI-EEG signals in $\beta$ band have similar recognition rates with the model order of 8 and 1 while classification rates reach the highest with the model order of 2, which suggests the frequency domain model obtained by Fourier transform of time domain model cannot truly reflect the variation characteristics of MI-EEG in frequency domain. Last but not least, the best frequency bands, *m* values and average classification rates with 10 × 10-fold CV for each subject are summarized in Table 1.

**Table 1:** Summarization of the best parameters for all subjects on Dataset A

Subjects	Best frequency bands	Best *m* values	Average classification rates with 10 × 10-fold (%)
‘k3b’	$\beta_{2}$(21–30 Hz)	2	100.00
‘k6b’	$\alpha$(8–13 Hz)	1	82.25
‘l1b’	$\alpha$(8–13 Hz)	1	99.75

#### Classification rates without frequency band selection

**Fig. 7:**
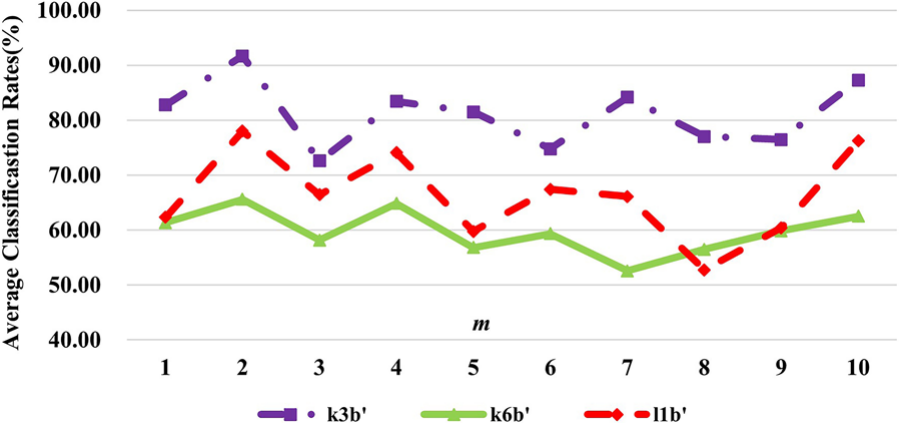
Effects of different *m* values on the classification results in $\alpha + \beta$ band for each subject

In this section, the classification rates without frequency band selection for 3 subjects are displayed in Fig. 7 for convenient observations.

By comparing the results in Fig. 7 with those in Figs. 5 and 6, we can find the best results without frequency band selection for subjects ‘k3b’, ‘k6b’ and ‘l1b’ are 91.67%, 65.58% and 78.00%, respectively, which are much lower than 100.00%, 82.25% and 99.75% with frequency band selection. It further verifies that adaptive selection of the best frequency band for individual subject can improve the classification rates. In the meantime, it is observed that the best classification accuracies are achieved when *m* value was set to 2 for all the three subjects and this phenomenon coincides with the above ones. What’s more, accuracies gain a certain degree of improvement ranging from 1.75 to 4.39% under selection of *m* values, which is lower than 8.33–17.06% under selection of both best frequency band and *m* values. This indicates that the double selection of the best frequency band and *m* values for each subject can yield the best results, which demonstrates the effectiveness of DDTF.

#### Visualizations of adjacency matrices, brain functional networks, outflows and inflows

It is noted from the results in Sects. 3.2.1 and 3.2.2 that recognition rates get promoted when *m* equals to 1 or 2 in any frequency band for each subject, so we take one trial of ‘k3b’ in $\alpha$ band for example to see the distinctions of adjacency matrices, brain functional networks, outflows and inflows under different *m* values. In addition, we denote ‘LH’ task as imagine left hand movement and ‘RH’ task as imagine right hand movement for convenient expressions.

**Fig. 8:**
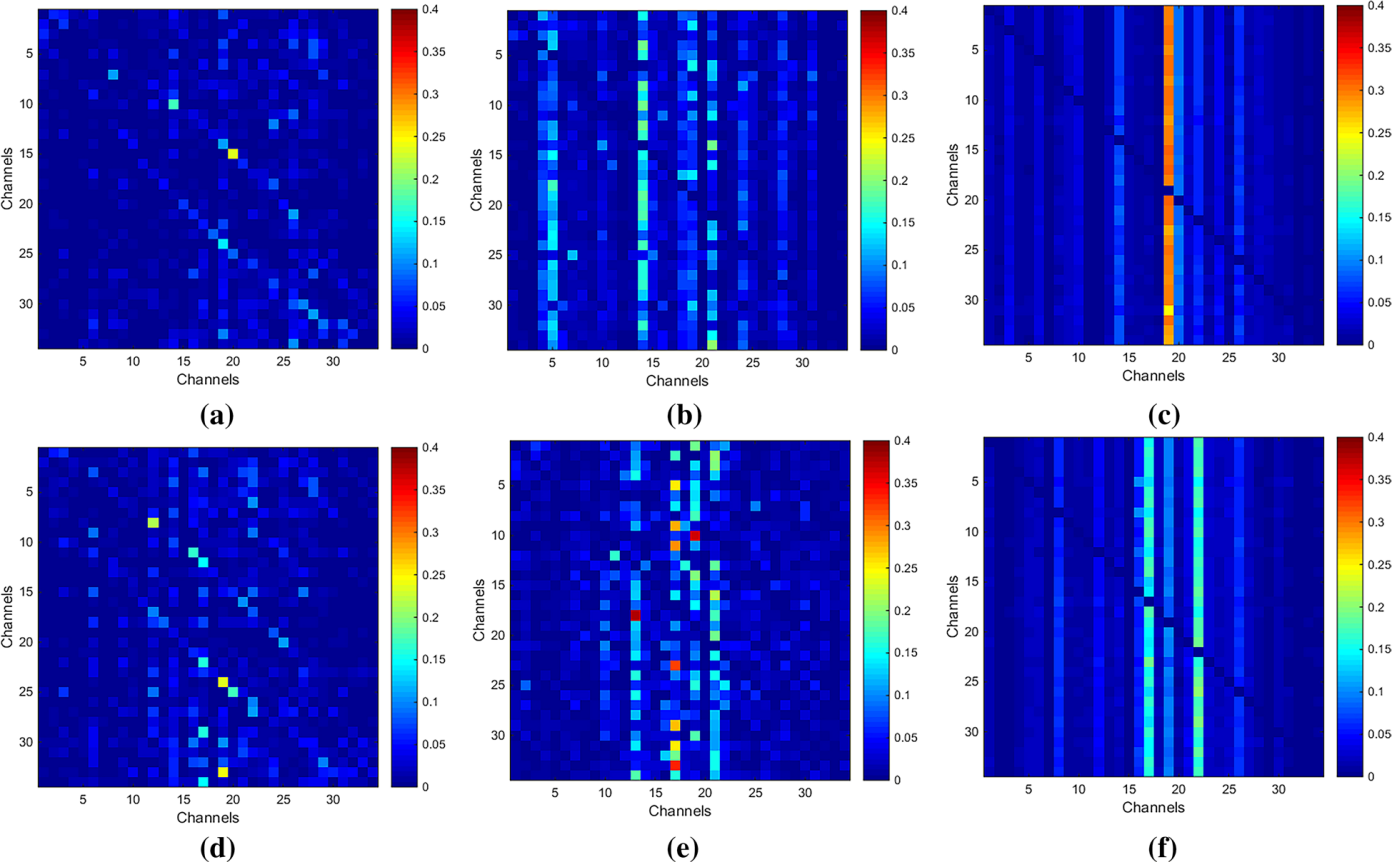
AMs of ‘k3b’ in $\alpha$ frequency band **a** *m* = 1 for ‘LH’ task, **b** *m* = 2 for ‘LH’ task, **c** *m* = 3 for ‘LH’ task, **d** *m* = 1 for ‘RH’ task, **e** *m* = 2 for ‘RH’ task and **f** *m* = 3 for ‘RH’ task

**Fig. 9:**
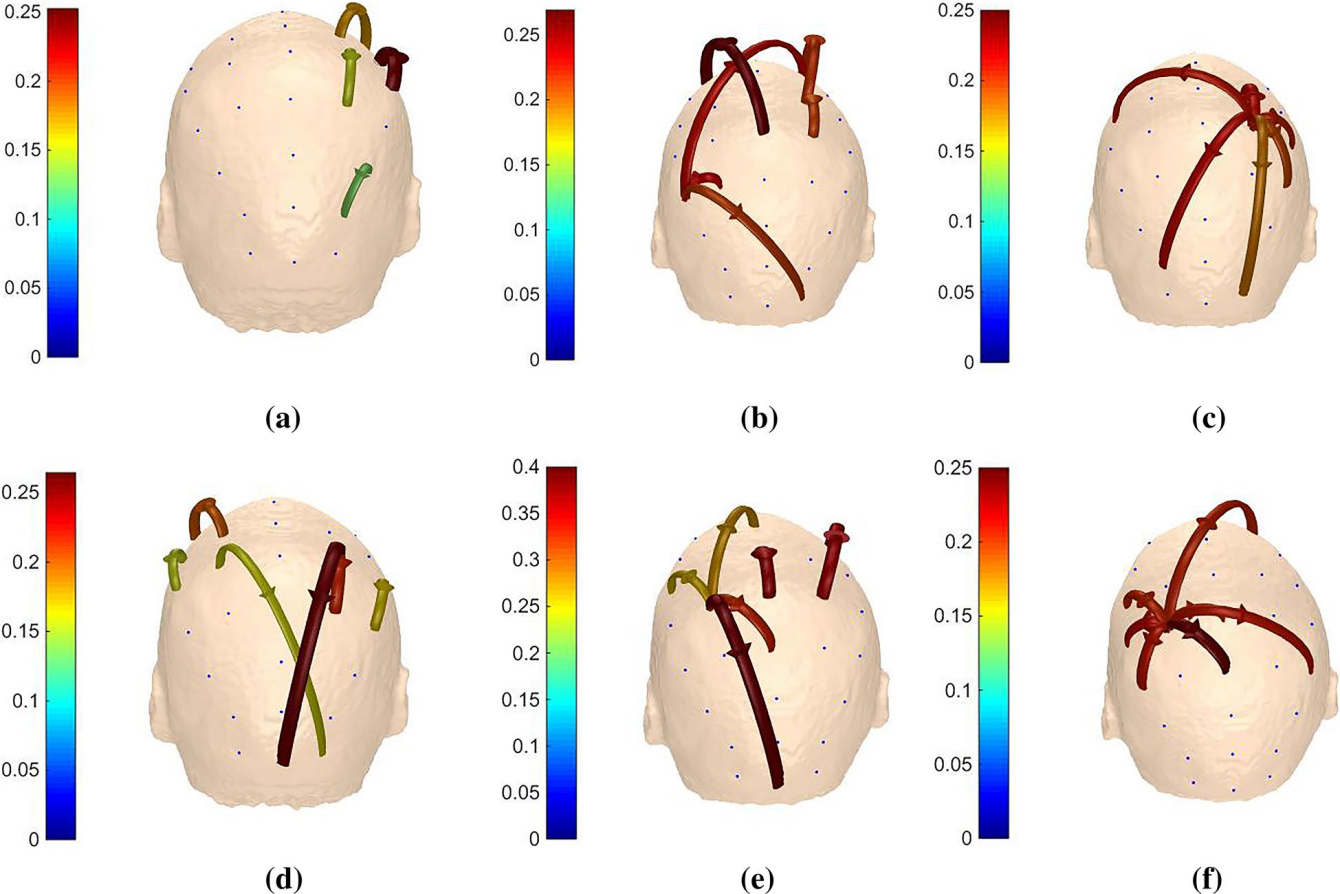
BFNs of ‘k3b’ in $\alpha$ frequency band **a** *m* = 1 for ‘LH’ task, **b** *m* = 2 for ‘LH’ task, **c** *m* = 3 for ‘LH’ task, **d** *m* = 1 for ‘RH’ task, **e** *m* = 2 for ‘RH’ task and **f** *m* = 3 for ‘RH’ task

The adjacency matrices under different *m* values for ‘LH’ and ‘RH’ tasks are expressed in Fig. 8. Horizontal and vertical axes represent the new channel number, elements in the matrices reveal the direction and strength of information between two channels. The closer the color is to red, the higher the intensity. On the contrary, the closer the color is to blue, the lower the intensity. Based on graph theory, the corresponding brain functional networks are constructed with EEG electrodes as nodes and the adjacency matrices as links between channels, as shown in Fig. 9. As shown in Figs. 8 and 9, channels located in the central parietal area, which are much more related with MI, have higher intensities. Conversely, channels far away from central parietal area have lower intensities because they are less related with motor activities.

**Fig. 10:**
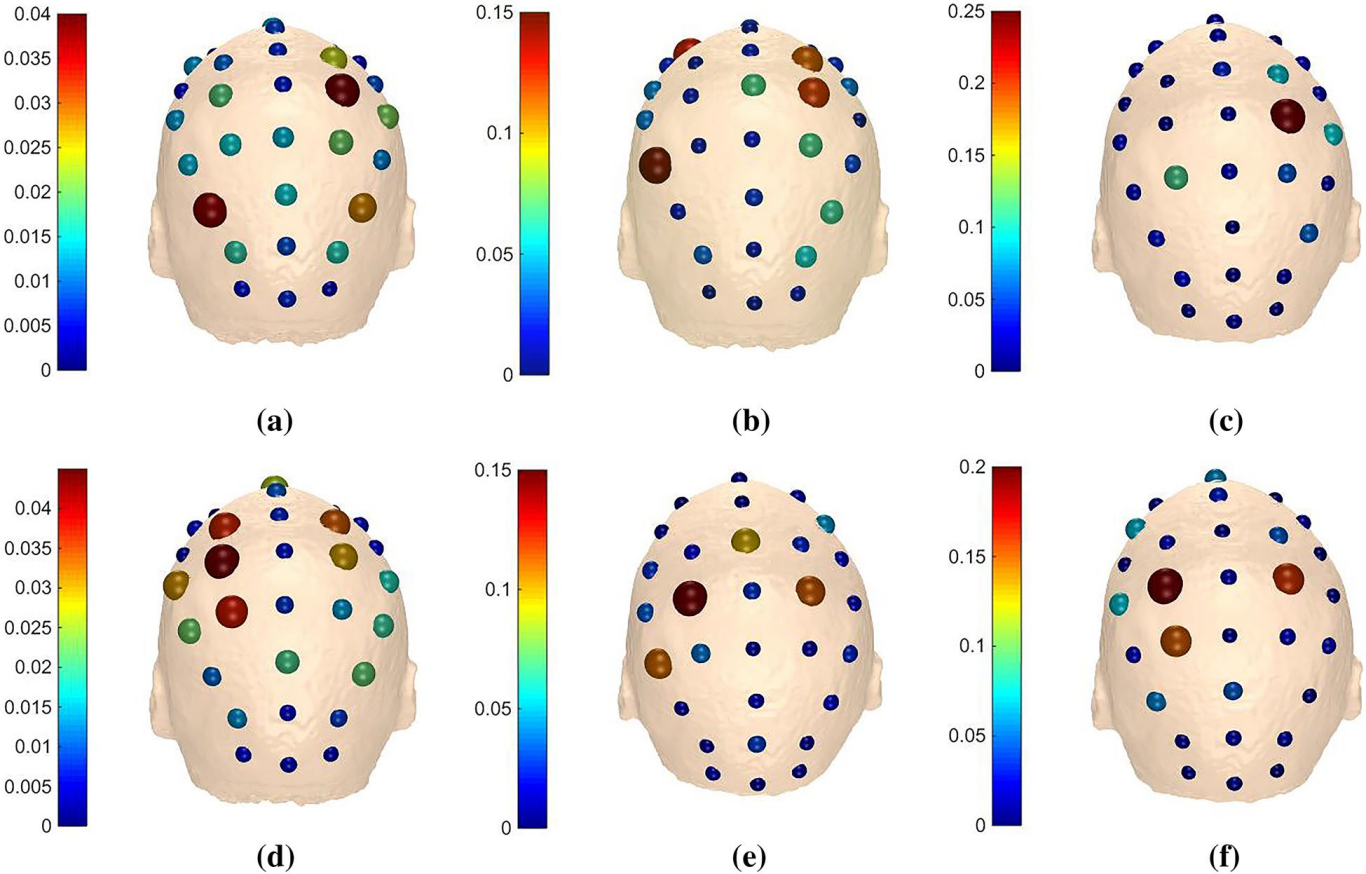
Outflows of ‘k3b’ in $\alpha$ frequency band **a** *m* = 1 for ‘LH’ task, **b** *m* = 2 for ‘LH’ task, **c** *m* = 3 for ‘LH’ task, **d** *m* = 1 for ‘RH’ task, **e** *m* = 2 for ‘RH’ task and **f** *m* = 3 for ‘RH’ task

**Fig. 11:**
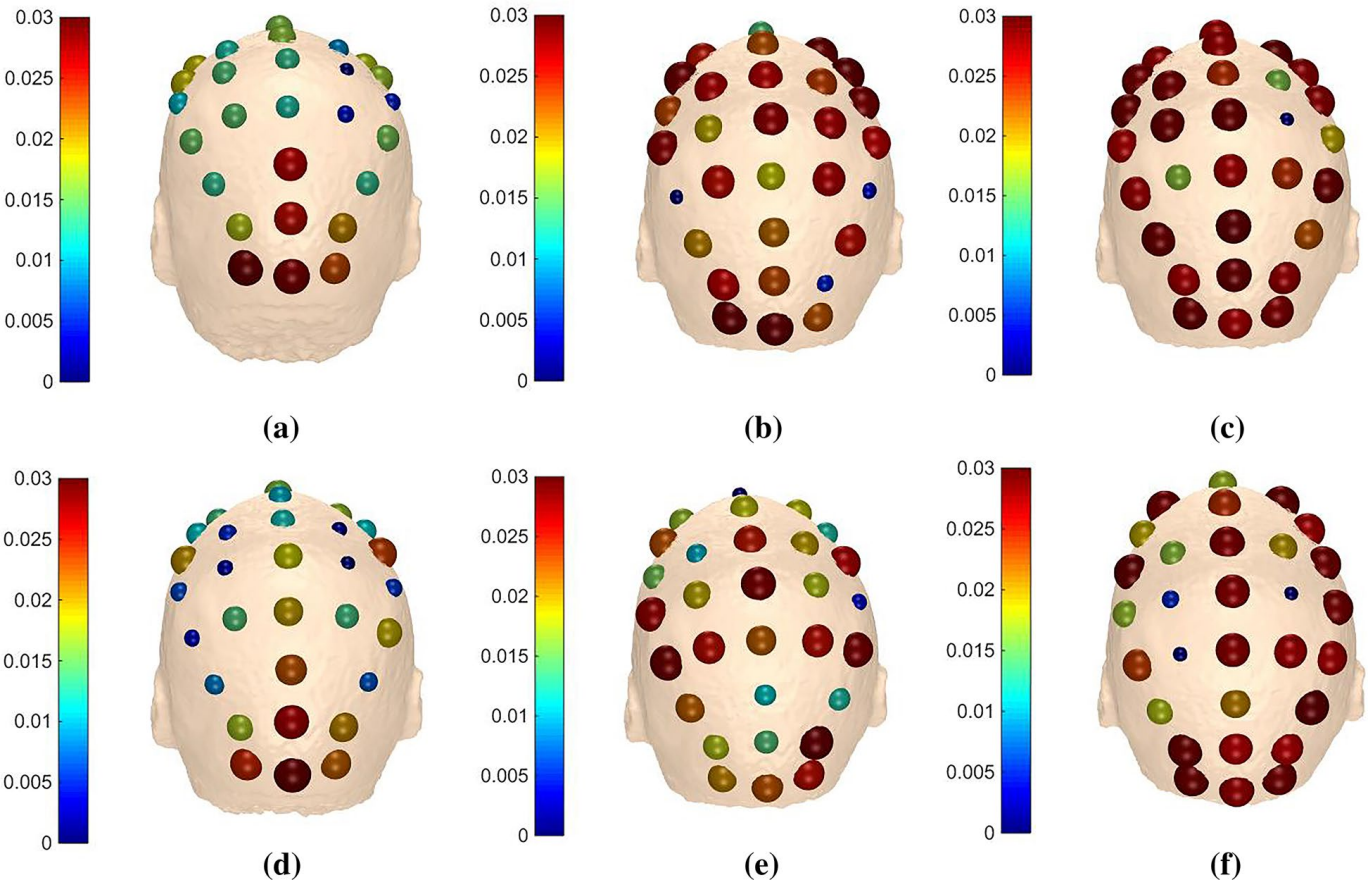
Inflows of ‘k3b’ in $\alpha$ frequency band **a** *m* = 1 for ‘LH’ task, **b** *m* = 2 for ‘LH’ task, **c** *m* = 3 for ‘LH’ task, **d** *m* = 1 for ‘RH’ task, **e** *m* = 2 for ‘RH’ task and **f** *m* = 3 for ‘RH’ task

**Fig. 12:**
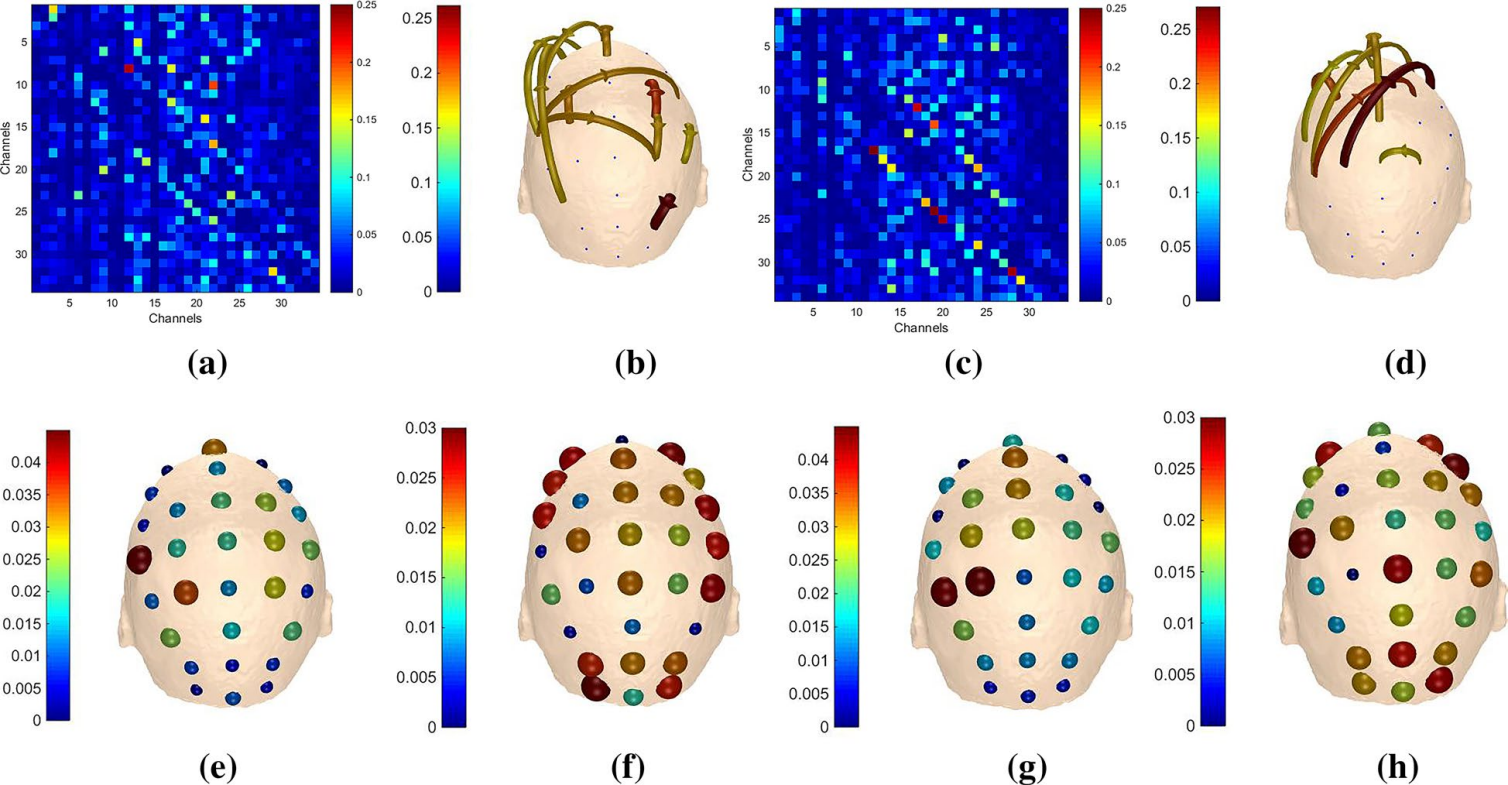
Adjacency matrices, brain functional networks, outflows and inflows of ‘k3b’ in ${\upbeta }$ frequency band **a** AM for ‘LH’ task, **b** BFN for ‘LH’ task, **c** AM for ‘RH’ task, **d** BFN for ‘RH’ task, **e** outflows for ‘LH’ task, **f** inflows for ‘LH’ task, **g** outflows for ‘RH’ task, **h** inflows for ‘RH’ task

Figures 10 and 11 demonstrate the outflows and inflows from all channels, respectively. Considering the spatial distribution of the networks, we can note from the figure that the electrodes located in the central parietal area, which are reported to be related with somatosensory and motor activity [45, 46], have larger outflows. Meanwhile, the contralateral primary sensorimotor area was activated during MI when one of the upper limbs was involved, information originates mainly from right parts of the brain when imaging left hand movement and vice versa. It is generally accepted that the movement of a body is denominated by the contralateral part of the brain. The adjacency matrices, brain functional networks, outflows and inflows in $\beta$ band of ‘k3b’ when *m* equals to 2 are also visualized in Fig. 12 for comparison. It can be observed that there are considerable differences between $\alpha$ and $\beta$ band, which further provides theoretical basis for our method.

### Experimental results on Dataset B

**Fig. 13:**
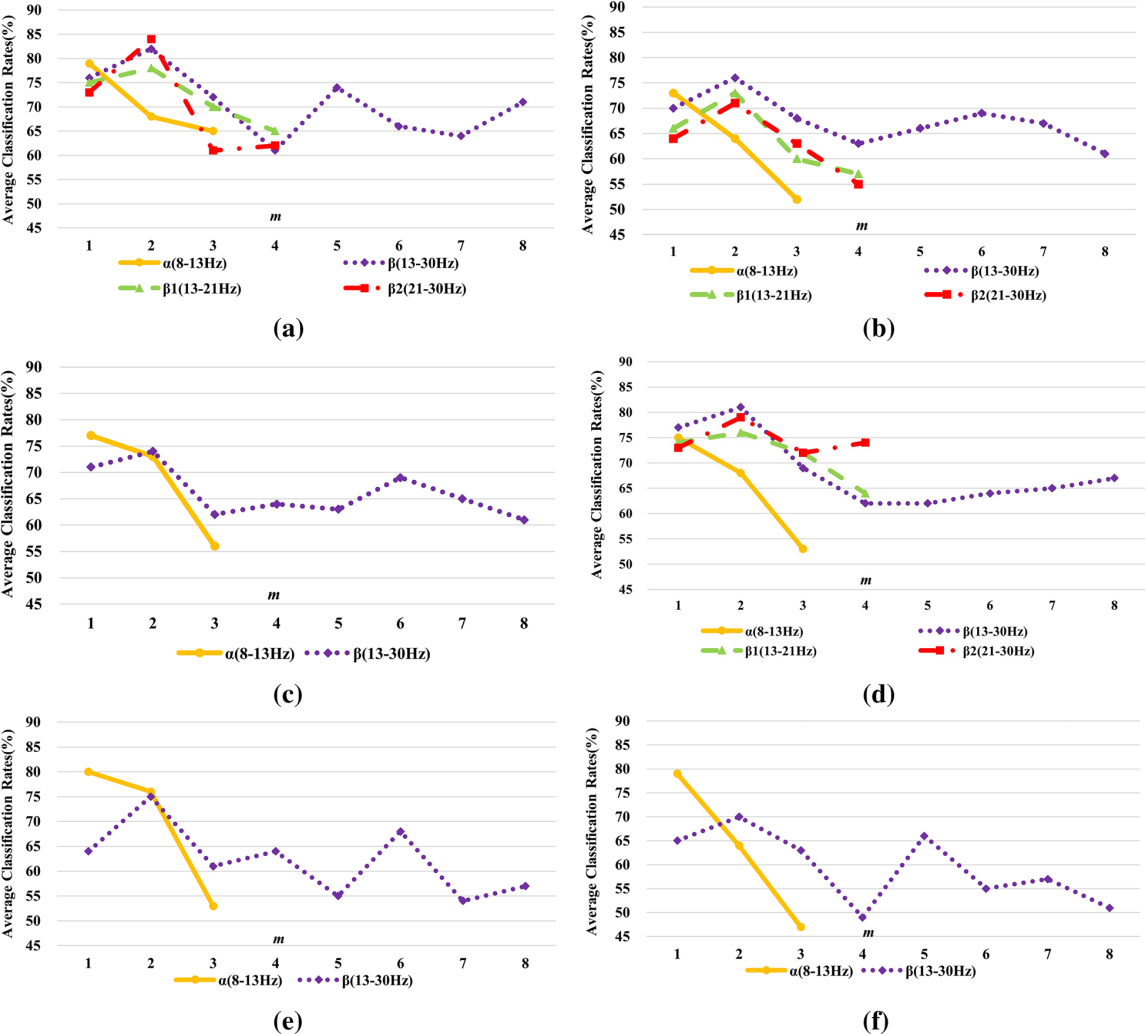

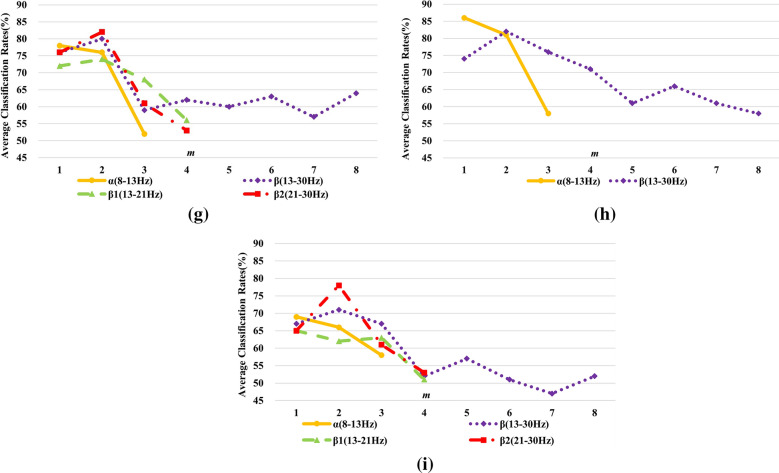
Effects of different frequency bands and *m* values on the classification results for **a** S1, **b** S2, **c** S3, **d** S4, **e** S5, **f** S6, **g** S7, **h** S8 and **i** S9 on Dataset B

Aiming a better insight of potential performance in real acquisition data, DDTF is extended to Dataset B. Under the same experimental procedure in Sect. 3.2, the 10 × 10-fold cross-validation classification accuracies are shown in Fig. 13 when DDTF is applied to extract features from 9 subjects, and the best parameters for each subject are summarized in Table 2. It can be found from Fig. 13 and Table 2 that the optimal frequency bands are varying for different subjects. Even for the same subject, the classification results over different frequency bands also have a significant disparity, which fully reflect the individual differences of MI-EEG. Therefore, the personalized selection of parameters will be helpful to improve the recognition rate for each subject. Not coincidentally, for any frequency band of all subjects, the recognition rates are greatly improved when *m* is equal to 1 or 2 instead of the original order *p*, which is consistent with the conclusion of Dataset A and further indicates the correctness and universality of DDTF.

**Table 2:** Summarization of the best parameters for all subjects on Dataset B

Subjects	Best frequency bands	Best *m* values	Average classification rates with 10 × 10-fold (%)
S1	$\beta_{2}$(21–30 Hz)	2	84
S2	$\beta$(13–30 Hz)	2	76
S3	$\alpha$(8–13 Hz)	1	77
S4	$\beta$(13–30 Hz)	2	81
S5	$\alpha$(8–13 Hz)	1	80
S6	$\alpha$(8–13 Hz)	1	79
S7	$\beta_{2}$(21–30 Hz)	2	82
S8	$\alpha$(8–13 Hz)	1	86
S9	$\beta_{2}$(21–30 Hz)	2	78

### Comparative experiments on Dataset A

#### Comparison with GC-based brain functional network methods

**Fig. 14:**
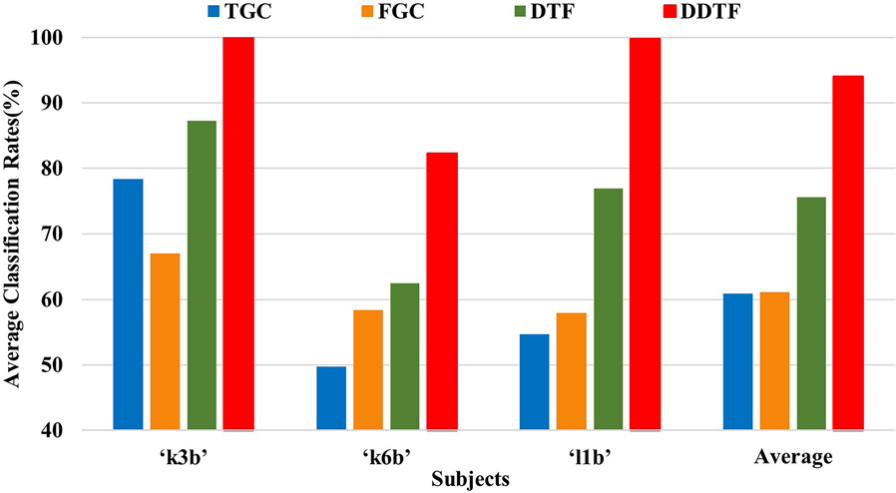
Comparison of average classification rates with GC-based BFN methods on Dataset A

To verify the validity of DDTF, some experiments were carried out to compare with GC-based brain functional network methods by which MI-EEG signals were filtered to 8–30 Hz and applied to construction of GC-based brain functional network, the extracted feature vectors were fed to SVM classifier. The average recognition results of 10 × 10-fold CV are displayed in Fig. 14.

From Fig. 14, it is easy to see that the classification performance resulted by Time-domain GC (TGC) is not ideal, this is mainly because this method only considers the interactions in time domain while ignoring the spectral properties of MI-EEG signals. Frequency-domain GC (FGC) uses both time and frequency information, which yields a slightly higher accuracies. However, these two methods just focus on information flow between two channels without considering the integrality of the whole brain. Despite the results of TGC and FGC being poor, DTF shows the advantages of multivariate autoregressive methods over traditional univariate methods for each subject in terms of classification accuracy. In addition, for each subject, the recognition rates obtained by using DDTF are significantly enhanced than those by using DTF. The time domain model and frequency domain model share the same model order in DTF, however, the frequency domain model cannot truly be used to construct and embody the changes of MI-EEG brain functional networks. Moreover, the individual differences of allied cognitive tasks are observed in DDTF, which produces better quality information. For all subjects, our method achieves relatively higher recognition accuracies than other GC-based methods, indicating the superiority of DDTF.

#### Statistical analysis

1. Kappa coefficient
  In this section, more statistical analyses were executed to confirm the effectiveness of DDTF. The kappa coefficient, which is generally thought to be a more robust measure than simple percent agreement calculation, takes into account the agreement occurring by chance. It is a common indicator for evaluating the performance of BCI systems [47]. The calculation of kappa coefficient is defined as:
  18 $$k = \frac{{p_{0} - p_{e} }}{{1 - p_{e} }},$$
  where $p_{0}$ indicates the classification accuracy, $p_{e}$ represents the probability of opportunity consistency. For a two-class task, if the number of samples across classes is equal, then the value of $p_{e}$ is 0.5. According to Eq. (18), the kappa coefficients of TGC, FGC, DTF as well as DDTF with 10 × 10-fold CV were calculated. The results are shown in Fig. 15.

**Fig. 15:**
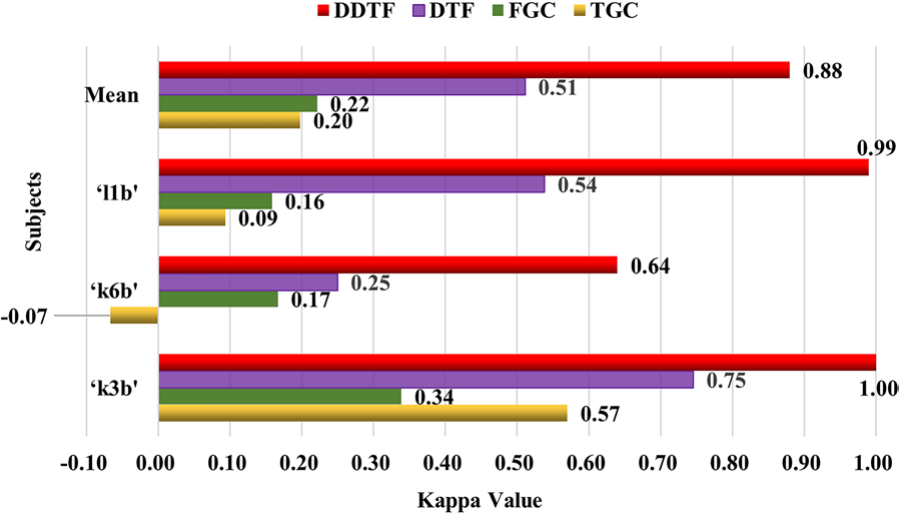
Comparison of Kappa coefficients with GC-based brain functional network methods
  Figure 15 indicates that DDTF achieves the highest kappa value among four methods for each subject. Especially compared to the original DTF, DDTF makes the kappa value increase by 0.39 for subject ‘k6b’ and 0.45 for subject ‘l1b’. Although there are variations in kappa values for different subjects, the mean kappa value of DDTF is improved by 0.37, 0.66 and 0.68 compared to DTF, FGC and TGC methods, respectively, which reveals that DDTF has better consistency in classification. Furthermore, the mean value of DDTF is 0.88, which is higher than 0.8, this illuminates a very good level of agreement.
2. *t*-Test
  To further analyze the differences between DTF and DDTF statistically, a two-sample *t*-test is proceeded to inspect whether there is a significant difference when they are available for MI-EEG feature extraction. Suppose that $\overline{M}_{{{\text{DTF}}}}$ and $\overline{M}_{{{\text{DDTF}}}}$ denote the mean values of tenfold CV accuracies generated by DTF and DDTF, similarly, $S^{2}_{{{\text{DTF}}}}$ and $S^{2}_{{{\text{DDTF}}}}$ stand for the variances, $n_{{{\text{DTF}}}}$ and $n_{{{\text{DDTF}}}}$ express the numbers of the results for the two methods, respectively. Then, the *t*-test statistic is calculated as follows:
  19 $$t = \frac{{\overline{M}_{{{\text{DDTF}}}} - \overline{M}_{{{\text{DTF}}}} }}{{\sqrt {\frac{{(n_{{{\text{DDTF}}}} - 1)S^{2}_{{{\text{DDTF}}}} + (n_{{{\text{DTF}}}} - 1)S^{2}_{{{\text{DTF}}}} }}{{n_{{{\text{DDTF}}}} + n_{{{\text{DTF}}}} - 2}}\left( {\frac{1}{{n_{{{\text{DDTF}}}} }} + \frac{1}{{n_{{{\text{DTF}}}} }}} \right)} }}.$$
  Define the null hypothesis is $H_{0}$: the results of DTF and DDTF originate from independent random samples from normal distributions with equal means; the alternative hypothesis is $H_{1}$: the results of DTF and DDTF come from populations with unequal means. The significance level is set as $\mu = 0.05$. The decision rule is to reject $H_{0}$, if:
  20 $$p = P\left\{ {t > t_{\mu } \left( {n_{{{\text{DDTF}}}} + n_{{{\text{DTF}} - 2}} } \right)} \right\} \le 0.05.$$
  It can be calculated that the values of *p* for 3 subjects are 2.0402 × 10^–16^, 3.0029 × 10^–22^ and 4.8337 × 10^–19^, respectively, and they are all less than 0.05. Hence, the null hypothesis $H_{0}$ is rejected at the 0.05 significance level, which implies DDTF outperforms DTF in MI-EEG feature extraction.

#### Comparison with multiple traditional feature extraction methods

CSP and its variants have been widely applied in feature extraction of MI-EEG and gained good recognition results based on BCI competition III Dataset. To further illustrate the feasibility of DDTF in this paper, the experiments were conducted to compare with multiple CSP-based methods in references [7–9, 47]. Table 3 illustrates the detailed information. It is clear that DDTF achieves the highest recognition rate of 100.00% and 99.75% for subjects ‘k3b’ and ‘l1b’, respectively, and the average classification accuracy, i.e., 94.00%, is superior to the best one with 91.87% in the CSP-based methods. The variants of CSP methods extract features in consideration of multi-channel and spatial distribution characteristics of MI-EEG signals while pitifully neglecting the relationships among EEG sensors. DDTF effectively excavates the interrelationship between multi-channel EEG signals, correctly analyzes the information exchange over the whole brain, and has better applicability in extracting features from MI-EEG signals.

**Table 3:** Comparison with multiple CSP-based feature extraction methods

Reference number	Methods	Subjects			Average accuracies (%)
		‘k3b’	‘k6b’	‘l1b’	
[7]	AC-CSP	97.80	63.30	94.20	85.10
[7]	AC-RCSP	97.80	72.50	95.00	88.43
[7]	CCS-CSP	98.90	79.20	95.80	91.30
[7]	CCS-RCSP	98.90	80.00	96.70	91.87
[8]	CSSSP	95.50	55.10	95.00	81.87
[8]	BCSP	78.80	63.70	76.60	73.03
[8]	ACSP	76.60	56.80	51.60	61.67
[8]	Pcv	**100.00**	68.90	96.60	88.50
[8]	Pfix	**100.00**	67.20	98.30	88.50
[9]	CSP	67.50	70.00	53.30	63.60
[9]	CCSP	95.00	**90.00**	83.30	89.43
This paper	DDTF	**100.00**	82.25	**99.75**	**94.00**

The bold values reflect the highest classification accuracies among all methods

Pcv and Pfix represent the CCSSP with and without automatic parameter selection, respectively

### Comparative experiments on Dataset B

**Fig. 16:**
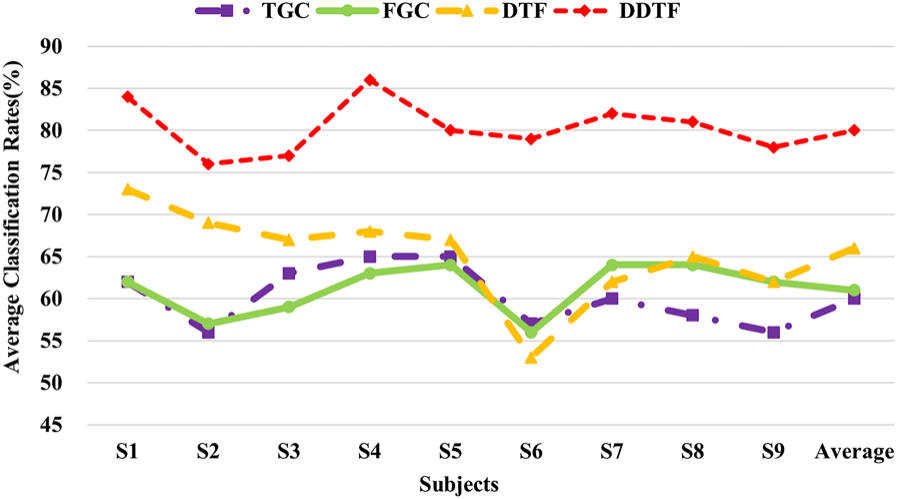
Comparison of average classification rates with GC-based BFN methods on Dataset B

In this section, DDTF was compared with the original DTF and GC-based BFN methods on Dataset B, and the results are shown in Fig. 16 and Table 4. It can be seen from Fig. 16 that for each subject, the classification accuracy of DTF is significantly higher than those of TGC, FGC and DTF when used for MI-EEG feature extraction. The average classification accuracy of DDTF increases by 14% compared to DTF, and S6 gets the highest, i.e., 26%. The Kappa coefficients in Table 4 shows that DDTF has an significant improvement in different degrees for 9 subjects, and the average Kappa value of DDTF is 0.61, which has an increase of 0.31, 0.41 and 0.38 relative to those of DTF, TGC and FGC, respectively, revealing that DDTF has the best consistency.

**Table 4:** Comparison of Kappa coefficients with GC-based BFN methods on Dataset B

Methods	Subjects									Average
	S1	S2	S3	S4	S5	S6	S7	S8	S9	
TGC	0.24	0.12	0.26	0.16	0.30	0.14	0.20	0.30	0.12	0.20
FGC	0.24	0.16	0.18	0.28	0.28	0.12	0.28	0.26	0.24	0.23
DTF	0.46	0.38	0.34	0.30	0.34	0.06	0.24	0.36	0.24	0.30
DDTF	0.68	0.52	0.54	0.62	0.60	0.58	0.64	0.72	0.56	0.61

## Discussion

**Fig. 17:**
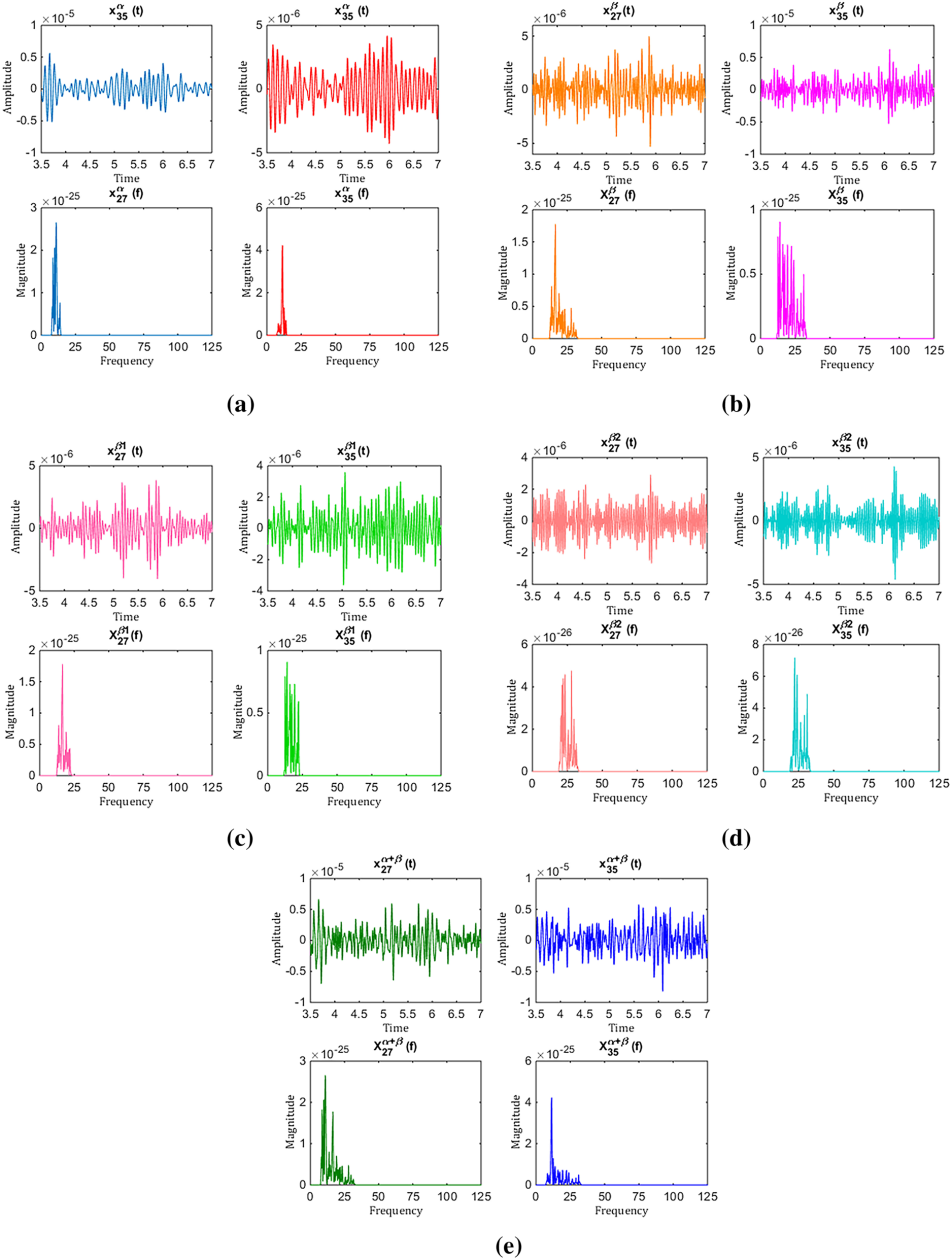
The variations of channels 27 and 35 in time and frequency domains filtered to **a** $\alpha$ band, **b** $\beta$ band, **c** $\beta_{1}$ band, **d** $\beta_{2}$ band and **e** $\alpha + \beta$ band

In this paper, DDTF was proposed for construction and feature extraction of brain functional network. In DDTF, the optimal frequency band is adaptively selected for each subject, which preferably detects the subject-based feature of MI-EEG, and ${\varvec{B}}^{m} \left( f \right)$ is defined with a varying order $m$, this is helpful to improve the classification rates. In addition, the best classification accuracy in any frequency band is achieved for each subject when *m* value equals to 1 or 2. To seek for the reasons, we took subject ‘k3b’ from Dataset A as an example and drew the changing curves of channels 27 and 35 MI-EEG signals in time and frequency domains, which are illustrated as Fig. 17. Figure 17a–e shows the variations of channels 27 and 35 in time and frequency domains when filtered to $\alpha ,$ $\beta ,$ $\beta_{1} ,$ $\beta_{2}$ and $\alpha + \beta$ frequency bands, respectively. As is known to all, MI-EEG signals are approximately stationary in short time intervals when the MVAR model is constructed in time domain. Therefore, the time domain MVAR model can express the changes of time domain signals correctly. However, the non-stationarity of frequency domain signals becomes very intense because of the activated characteristics generated by motor imagery in either ${\upalpha }$ or ${ }\beta { }$ frequency band, which can be clearly seen from Fig. 17. Although DTF can correctly reflect the quantitative relationships between the time and frequency domain model, it may not effectively express the characteristics of the frequency domain MI-EEG signals. Therefore, we built the frequency domain MVAR model with model order of 1 based on the MI-EEG filtered to $\alpha { }$ band, the results perfectly match with the non-stationarity of MI-EEGs in frequency domain. In [26–28], the time domain model and frequency domain model share the same model order, which ignores or weakens the non-stationarity of frequency domain signals. Particularly, DDTF is the same as DTF [26–28] when $m_{\alpha }$ equals to $p_{\alpha }$ while DDTF can represent the variation characteristics of MI-EEG in frequency domain and the constructed brain functional networks are more veritable and objective. The same experiments were also carried on other subjects and we got similar conclusions.

The adjacency matrices and brain functional networks in Figs. 8 and 9 show that with the increase of *m* values, information transfers get sharp augments and brain functional networks are in the transition from transient state to stable state. Figure 10 indicates the electrodes with larger outflows are located in the central parietal area which are related to motor activity, and the information originates mainly from right part of the brain when imaging left hand movement and vice versa, which is in accordance with the well-accepted theory. What is said above further provides theoretical support for our method.

In addition, DDTF were compared with GC-based BFN feature extraction methods, and DDTF achieved the highest average classification accuracies based on two Datasets and 12 subjects, demonstrating its effectiveness in discrimination of motor imagery tasks, as shown in Figs. 14 and 16. Robustness test was also performed on both Datasets A and B by using Kappa coefficient, the results are shown in Fig. 15 and Table 4. It means that the proposed DDTF achieved the relatively higher Kappa values and even got greatly improvement for each subject compared to GC-based methods and original DTF. This indicates DDTF has good adaptive ability to different subjects, the main reason may be that DDTF can dynamically determine the specific model order and related frequency bands according to the cortical activation induced by motor imagery. Besides, a two-sample *t*-test statistic was designed to explore whether there was a significant difference between DTF and DDTF in MI-EEG feature extraction. The results implied the superiority and feasibility of DDTF in brain functional network-based feature extraction. This provided a new idea for extracting the features of MI-EEG as well as enhancing the adaptivity of feature extraction.

## Conclusions

A dynamic DTF, called DDTF, was developed in this study. It is calculated based on a dynamic frequency domain model with a lower order, and only the most related information is beneficial for recognition of MI-EEG, i.e., the previous 1 or 2 moment is much more related to the current state, and the brain functional network changes from transient state to stable state with the increment of model order. Meanwhile, the best frequency band can be adaptively sought for each individual subject. This makes it more closely coincident with the subject-based characteristic of MI-EEG, yielding better features and recognition rates. Extended experimental results have suggested that DDTF achieves excellent performance in brain functional network-based feature extraction. In the future study, we intend to integrate the DDTF-based brain functional network with other feature extraction methods to improve its property. In addition, it will provide a good prospect for disease diagnosis, seizure detection and rehabilitation effect evaluation.

## Data Availability

The Dataset A generated and analyzed during the current study is available in the BCI Competition III Dataset IIIa repository, http://www.bbci.de/competition/iii. The Dataset B used and analyzed during the current study is available from the corresponding author on reasonable request.

## References

[1] Wolpaw JR, Birbaumer N, McFarland DJ (2002). Brain–computer interfaces for communication and control. Clin Neurophysiol.

[2] Ang KK, Guan C (2016). EEG-based strategies to detect motor imagery for control and rehabilitation. IEEE Trans Neur Syst Rehabilit Eng.

[3] Li M, Xi H, Sun Y (2019). Feature extraction and visualization of MI-EEG with L-MVU algorithm. World congress on medical physics and biomedical engineering.

[4] Blankertz B, Müller KR, Curio G (2004). The BCI competition 2003. IEEE Trans Biomed Eng.

[5] Blankertz B, Muller KR, Krusienski DJ (2006). The BCI competition III: validating alternative approaches to actual BCI problems. IEEE Trans Neur Sys Rehabilit Eng.

[6] Falzon O, Camilleri KP, Muscat J (2010) Complex-valued spatial filters for task discrimination. In: 2010 annual international conference of the IEEE engineering in medicine and biology, IEEE, pp 4707–4710 10.1109/IEMBS.2010.5626381 21096013

[7] Jin J (2019). Correlation-based channel selection and regularized feature optimization for MI-based BCI. Neural Netw.

[8] Yu K, Wang Y, Shen K (2013). The Synergy between complex channel-specific FIR filter and spatial filter for single-trial EEG classification. PLoS ONE.

[9] Li L, Xu G, Xie J (2019). Classification of single-trial motor imagery EEG by complexity regularization. Neural Comput Appl.

[10] McEvoy LK, Smith ME, Gevins A (1998). Dynamic cortical networks of verbal and spatial working memory: effects of memory load and task practice. Cereb Cortex.

[11] Li F, Tian Y, Zhang Y (2015). The enhanced information flow from visual cortex to frontal area facilitates SSVEP response: evidence from model-driven and data-driven causality analysis. Sci Rep.

[12] Li F, Liu T, Wang F (2015). Relationships between the resting-state network and the P3: evidence from a scalp EEG study. Sci Rep.

[13] Zhang Y, Xu P, Guo D (2013). Prediction of SSVEP-based BCI performance by the resting-state EEG network. J Neural Eng.

[14] Zhang T, Liu T, Li F (2016). Structural and functional correlates of motor imagery BCI performance: insights from the patterns of fronto-parietal attention network. Neuroimage.

[15] Li F, Chen B, Li H (2016). The time-varying networks in P300: a task-evoked EEG study. IEEE Trans Neur Syst Rehabilit Eng.

[16] Wang G, Sun Z, Tao R (2015). Epileptic seizure detection based on partial directed coherence analysis. IEEE J Biomed Health Inform.

[17] Van Mierlo P, Papadopoulou M, Carrette E (2014). Functional brain connectivity from EEG in epilepsy: seizure prediction and epileptogenic focus localization. Prog Neurobiol.

[18] Takigawa M, Wang G, Kawasaki H (1996). EEG analysis of epilepsy by directed coherence method a data processing approach. Int J Psychophysiol.

[19] Wang J, Wang X, Xia M (2015). GRETNA: a graph theoretical network analysis toolbox for imaging connectomics. Front Hum Neurosci.

[20] Friston KJ (1994). Functional and effective connectivity in neuroimaging: a synthesis. Hum Brain Mapp.

[21] Ahmadi N, Pei Y, Carrette E (2020). EEG-based classification of epilepsy and PNES: EEG microstate and functional brain network features. Brain Inform.

[22] Yao Z, Hu B, Xie Y (2015). A review of structural and functional brain networks: small world and atlas. Brain Inform.

[23] Wiener N (1956). The theory of prediction. Modern mathematics for engineers.

[24] Granger CWJ (1969). Investigating causal relations by econometric models and cross-spectral methods. Econom J Econom Soc.

[25] Geweke J (1982). Measurement of linear dependence and feedback between multiple time series. J Am Stat Assoc.

[26] Bastos AM, Schoffelen JM (2016). A tutorial review of functional connectivity analysis methods and their interpretational pitfalls. Front Syst Neurosci.

[27] Kaminski MJ, Blinowska KJ (1991). A new method of the description of the information flow in the brain structures. Biol Cybern.

[28] Kamiński M, Ding M, Truccolo WA (2001). Evaluating causal relations in neural systems: Granger causality, directed transfer function and statistical assessment of significance. Biol Cybern.

[29] Ding M, Bressler SL, Yang W (2000). Short-window spectral analysis of cortical event-related potentials by adaptive multivariate autoregressive modeling: data preprocessing, model validation, and variability assessment. Biol Cybern.

[30] Ginter J, Blinowska KJ, Kaminski M et al (2005) Propagation of brain electrical activity during real and imagined motor task by directed transfer function. In: Conference proceedings 2nd international IEEE EMBS conference on neural engineering, IEEE, pp 105–108

[31] Yi W, Zhang L, Wang K et al (2014) Evaluation and comparison of effective connectivity during simple and compound limb motor imagery. In: 2014 36th annual international conference of the IEEE engineering in medicine and biology society, IEEE, pp 4892–4895 10.1109/EMBC.2014.6944720 25571088

[32] Korzeniewska A, Mańczak M, Kamiński M (2003). Determination of information flow direction among brain structures by a modified directed transfer function (dDTF) method. J Neurosci Methods.

[33] Billinger M, Brunner C, Müller-Putz GR (2013). Single-trial connectivity estimation for classification of motor imagery data. J Neural Eng.

[34] Heger D, Terziyska E, Schultz T (2014) Connectivity based feature-level filtering for single-trial EEG BCIs. In: 2014 IEEE international conference on acoustics, speech and signal processing (ICASSP), IEEE, pp 2064–2068

[35] Wilke C, Ding L, He B (2008). Estimation of time-varying connectivity patterns through the use of an adaptive directed transfer function. IEEE Trans Biomed Eng.

[36] Li F, Peng W, Jiang Y (2019). The dynamic brain networks of motor imagery: time-varying causality analysis of scalp EEG. Int J Neural Syst.

[37] Wang D, Ren D, Li K (2018). Epileptic seizure detection in long-term EEG recordings by using wavelet-based directed transfer function. IEEE Trans Biomed Eng.

[38] Vanhatalo S, Voipio J, Kaila K (2005). Full-band EEG (fbEEG): a new standard for clinical electroencephalography. Clin EEG Neurosci.

[39] Millan JR (2004) On the need for on-line learning in brain–computer interfaces. In: 2004 IEEE international joint conference on neural networks (IEEE Cat. No. 04CH37541), vol 4. IEEE, pp 2877–2882

[40] Wu J, Srinivasan R, Kaur A (2014). Resting-state cortical connectivity predicts motor skill acquisition. Neuroimage.

[41] Knight RT (2007). Neural networks debunk phrenology. Science.

[42] Schwarz G (1978). Estimating the dimension of a model. Ann Stat.

[43] BCI Competition III (2005) Graz University of Technology. http://www.bbci.de/competition/iii

[44] Santamaria L, James C (2016) Use of graph metrics to classify motor imagery based BCI. In: 2016 international conference for students on applied engineering (ICSAE), IEEE, pp 469–474

[45] Angulo-Sherman IN, Gutiérrez D (2015). A link between the increase in electroencephalographic coherence and performance improvement in operating a brain–computer interface. Comput Intel Neurosci.

[46] Babiloni C, Brancucci A, Vecchio F (2006). Anticipation of somatosensory and motor events increases centro-parietal functional coupling: an EEG coherence study. Clin Neurophysiol.

[47] She Q, Ma Y, Meng M (2015). Multiclass posterior probability twin svm for motor imagery EEG classification. Comput Intel Neurosci.

